# A Quadruped Robot Exhibiting Spontaneous Gait Transitions from Walking to Trotting to Galloping

**DOI:** 10.1038/s41598-017-00348-9

**Published:** 2017-03-21

**Authors:** Dai Owaki, Akio Ishiguro

**Affiliations:** 1 Research Institute of Electrical Communication, Tohoku University, Katahira 2-1-1, Aoba-ku, Sendai 980-8577 Japan; 2 CREST, Japan Science and Technology Agency, 4-1-8 Honcho, Kawaguchi, Saitama 332-0012 Japan

## Abstract

The manner in which quadrupeds change their locomotive patterns—walking, trotting, and galloping—with changing speed is poorly understood. In this paper, we provide evidence for interlimb coordination during gait transitions using a quadruped robot for which coordination between the legs can be self-organized through a simple “central pattern generator” (CPG) model. We demonstrate spontaneous gait transitions between energy-efficient patterns by changing only the parameter related to speed. Interlimb coordination was achieved with the use of local load sensing only without any preprogrammed patterns. Our model exploits *physical communication* through the body, suggesting that knowledge of physical communication is required to understand the leg coordination mechanism in legged animals and to establish design principles for legged robots that can reproduce flexible and efficient locomotion.

## Introduction

**Figure 1:**
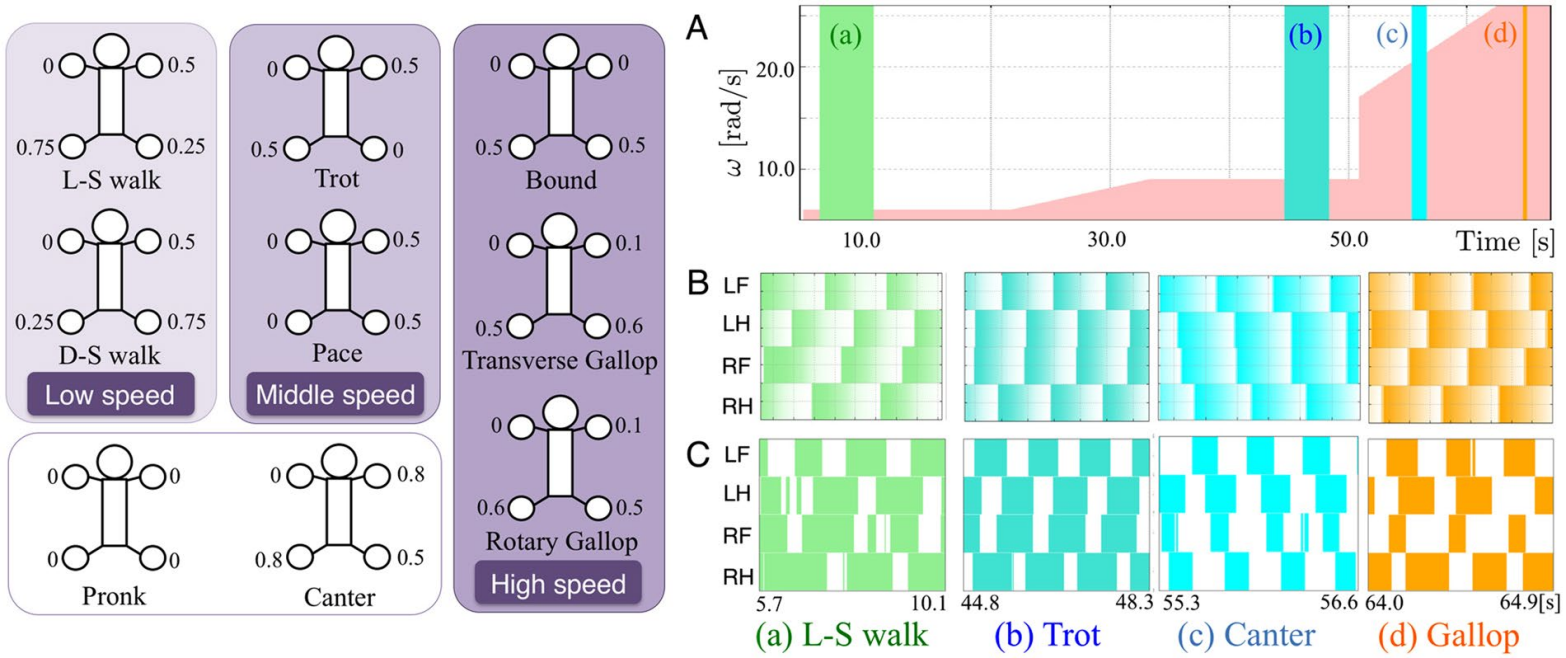
Gait patterns in quadruped animals^2^. The values shown around the feet represent the typical phases relative to the left fore (LF) leg. (**A**) Gait transition experiment. Profile of parameter *ω*. We used the parameter value *σ* = 6.0 [rad/Ns]. An abrupt change in *ω* at t = 50.6 s corresponds to the jump over the unstable gap between the trot and gallop gaits (see the instability in Fig. 3(C) at *ω* = 12.0, 14.0. We explain the reason for this in detail in the SM). (**B**) Evolution of the oscillator phase in each leg. The coloured shading represents the phase value (color: *ϕ*_*i*_ = 0, white: *ϕ*_*i*_ = 2*π*). (**C**) Gait diagrams. The coloured regions represent the stance phase, during which the sensor value *N*_*i*_ becomes greater than a threshold value (10% of maximum pressure).

Quadrupeds, or four-legged animals, were the first vertebrates able to move on Earth by using their legs. Because of the dramatic evolutionary changes from underwater to terrestrial environments over time, four-legged animals evolved to counteract the effect of gravity, negotiate terrestrial ground, and locomote more efficiently for predation and survival. By coordinating leg movements, i.e.“interlimb coordination”, quadrupeds can change their locomotive patterns, e.g. walking, trotting, and galloping (Fig. 1) to adopt the most energy-efficient gait for a given speed^1, 2^. Thus, knowledge of the interlimb coordination mechanism underlying quadruped gait transition is essential for understanding the locomotive mechanism in legged animals and is useful for establishing design principles for legged robots that can reproduce flexible and efficient locomotion.

Autonomous decentralized control is a key concept in the interlimb coordination mechanism. Past experiments involving decerebrate cats^3^ have suggested that their locomotive patterns are controlled in part by a distributed neural network called the “central pattern generator” (CPG) in the spinal cord^4, 5^. These findings have sparked a surge in interdisciplinary research to model an intraspinal neural circuit of this sort and reproduce the relevant locomotive patterns^6–11^. However, no past study based on CPG has succeeded in reproducing gait transitions from low- to high-speed locomotion using a quadruped robot. This is because most relevant research has been either completely or partly based on preprogrammed neural network topologies in CPGs, and therefore has not involved “embodiment”^12^ to a sufficient degree, leading to pattern generation that neither self-organizes nor is efficient in response to real-world scenarios.

From another distinct control paradigm, i.e. a purely sensory-driven system, previous works have investigated the functional role of sensory feedback in quadruped limb coordination^13^ as well as human^14^ and insect walking^15, 16^. Neuro-mechanical approaches using computer simulations have indicated that locomotion can emerge from the interaction between sensory feedback signals and the body alone without CPG. This suggests that embodiment plays an essential role in structuring sensory information from the mechanical interaction of the body with the environment for flexible limb coordination. However, owing to sensitivity to sensory noise or sensory failure^17^, the validity of a purely sensory-driven system in real-world environments remains unclear.

To address the above issues, we use *embodied synthesis*, a synthetic approach grounded in embodiment, to understand the mechanisms underlying animal locomotion by building a physical robot that can move in the real world^18, 19^. This approach has two advantages: (i) we can test such a robot in environments similar to those encountered by animals without the need to model the environments, thus allowing for sound evaluation of their performance, e.g. in terms of efficiency; and (ii) we can design a minimal robot by simplifying its musculoskeletal and neural systems, allowing for extraction of sufficient conditions to explain the underlying mechanism of interest. Here, using a simple quadruped robot, we particularly address two essential issues related to quadruped gait transition: (I) Could transition between gaits be achieved as the result of a self-organization process driven by our simple CPG model^20^?; and (II) Could a single, velocity-related command variable be used to drive naturalistic gait transitions in our robot? Biological data suggest the existence of a velocity-related descending signal from the mesencephalic locomotor region (MLR) in the midbrain^21^. In this paper, we show the manner in which leg coordination can be self-organized through our CPG model, with spontaneous gait transitions between the most energy-efficient patterns exhibited only by changing the intrinsic angular velocity of oscillators in the CPG model without any preprogrammed gait patterns. Our model exploits *physical communication* through embodiment, suggesting that a local load-sensing mechanism is essential for speed-dependent gait transition in quadrupeds.

## Results

### Gait Transition

Our quadruped robot reproduced spontaneous gait transitions from a lateral-sequence (L-S) walk to a trot and then to a gallop in response to the locomotion speed (Movie S1). The MLR signal output increases the magnitude of the parameter of intrinsic angular velocity *ω* of a oscillator in each leg of our CPG model^20^ (Eq. 1). We conducted experiments using a treadmill and hand-tuned the treadmill’s speed by changing the value of *ω* (Fig. 1(A)). At *ω* = 6.0 [rad/s] (see part (a) of Fig. 1(A)), our robot exhibited an L-S walk in which the feet touched the ground in the following order: right hind (RH) leg, right fore (RF) leg, left hind (LH) leg and left fore (LF) leg. At *ω* = 9.0 [rad/s] (see part (b) of Fig. 1(A)), a trot was observed, with the diagonal feet touching the ground in phase. At *ω* = 26.0 [rad/s] (see part (d) of Fig. 1(A)), a gallop was observed, with the fore/hind feet landing on the ground in phase. Surprisingly, we observed a “canter” —an asymmetric three-beat gait—between 55.3 and 56.6 [s] from the start of the experiment (see part (c) of Fig. 1(A)). This gait is typically observed during the transition from trot to gallop in a horse^22^.

### Interlimb Coordination Analysis

**Figure 2:**
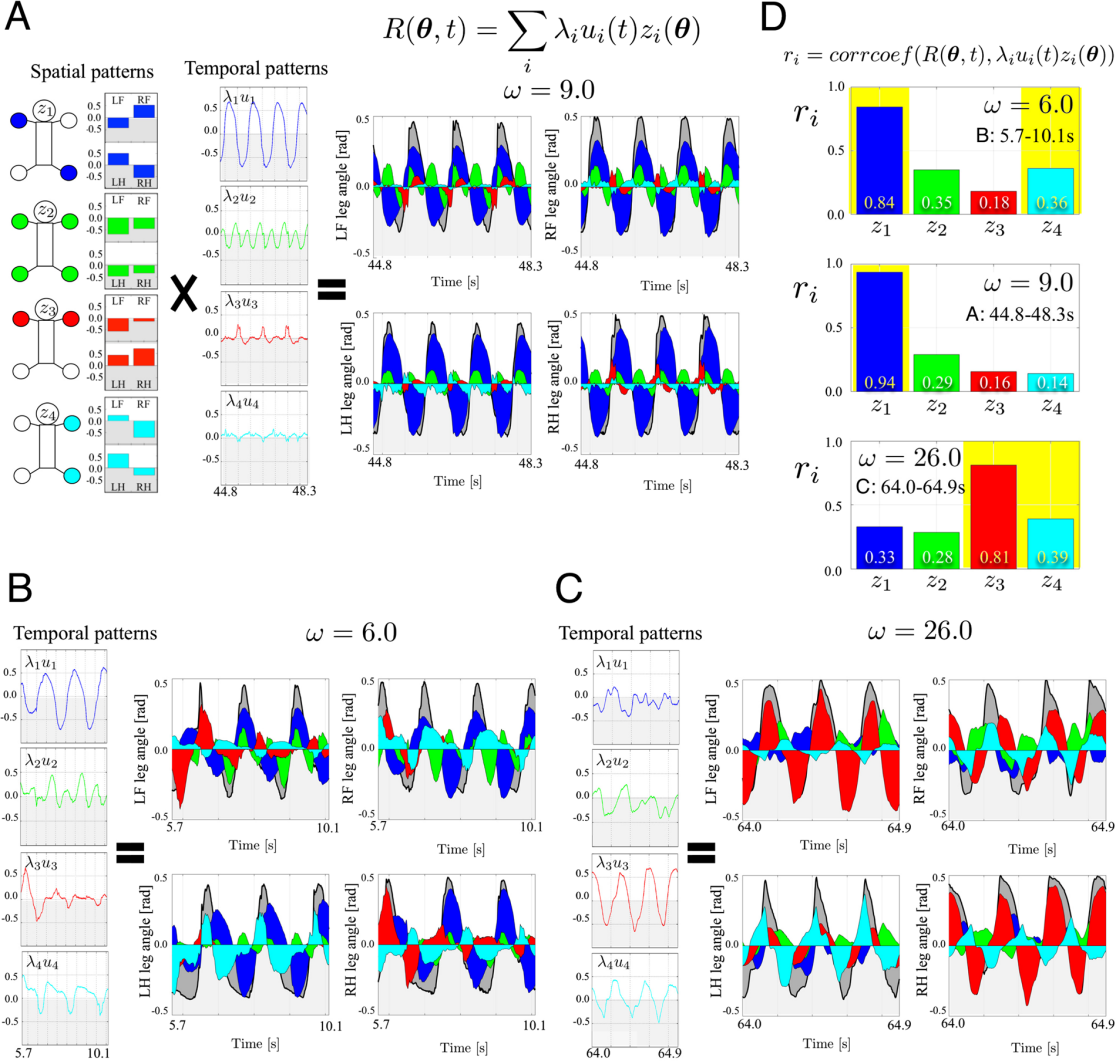
Interlimb coordination analysis. (**A**) Decomposition of data into time-invariant spatial patterns *z*_*i*_(***θ***) (left panels) and their temporal patterns *λ*_*i*_ *u*_*i*_(*t*) (middle panels) using SVD on four leg angles *R*(***θ***, *t*) (right panel). This gait is a trot (b) *ω* = 9.0 rad/s in Fig. 1(A). The grey, blue, green, red, and cyan areas in the right figure represent the values of *R*(***θ***, *t*), *λ*_1_ *u*_1_(*t*)*z*_1_(***θ***), *λ*_2_ *u*_2_(*t*)*z*_2_(***θ***), *λ*_3_ *u*_3_(*t*)*z*_3_(***θ***), and *λ*_4_ *u*_4_(*t*)*z*_4_(***θ***), respectively. In this gait, the spatial pattern *z*_1_ (blue, trotting) was the primary patten which sufficiently reproduced *R*(***θ***, *t*). The spatial patterns *z*_*i*_(***θ***) are common during gait transition experiments owing to their time-invariance. (**B**) and (**C**) depict the results for walking (a) *ω* = 6.0 rad/s and galloping (d) *ω* = 26.0 rad/s in Fig. 1(A), respectively. (**D**) Correlation analysis by calculating correlation coefficients *r*_*i*_ between *R*(***θ***, *t*) and *λ*_*i*_ *u*_*i*_(*t*)*z*_*i*_(***θ***). Walking (**B**) consisted primarily of the trot (blue) and pace (cyan) patterns, and galloping (**C**) consisted primarily of the bound (red) and pace (cyan) patterns. *ω*-dependent changes in the correlation coefficients of these patterns resulted in autonomous gait transition.

To analyse the interlimb coordination mechanism underlying the reproduced gait transition, we recorded three-dimensional (3-D) kinematic data, e.g. leg angles, using a real-time motion capture system. To decode the mechanism underlying gait transition, we used a decomposition approach for movement patterns^23–26^. Here, we calculated the time-invariant spatial patterns *z*_*i*_(***θ***) and their temporal patterns *λ*_*i*_ *u*_*i*_(*t*) as shown in Fig. 2 by applying singular value decomposition (SVD) to the four leg angles *R*(***θ***, *t*) = [*θ*_*LF*_(*t*), *θ*_*LH*_(*t*), *θ*_*RF*_(*t*), *θ*_*RH*_(*t*)] (see the ‘Methods’ section for further details). The results indicated that the gait patterns consisted of the four well-known basic time-invariant spatial patterns (Fig. 2(A) left), i.e. trotting (blue), pronking (green, with all feet touching the ground in phase), bounding (red, where fore and hind feet touching the ground half a period out of phase), and pacing (cyan, a two-beat gait in which the lateral feet land on the ground in phase). Furthermore, based on correlation analysis achieved by calculating the correlation coefficient values *r*_*i*_ between *R*(***θ***, *t*) and their four components *λ*_*i*_ *u*_*i*_(*t*)*z*_*i*_(***θ***) in Fig. 2(D), we found the following results: the L-S walk (B) consisted primarily of the trot (blue) and pace (cyan) patterns and the gallop (C) consisted of the bound (red) and pace (cyan) patterns. These patterns closely resembled those predicted from group theory^27^. Such *ω*-dependent changes in the correlation coefficients of these patterns resulted in autonomous gait transition.

### Energy Efficiency

**Figure 3:**
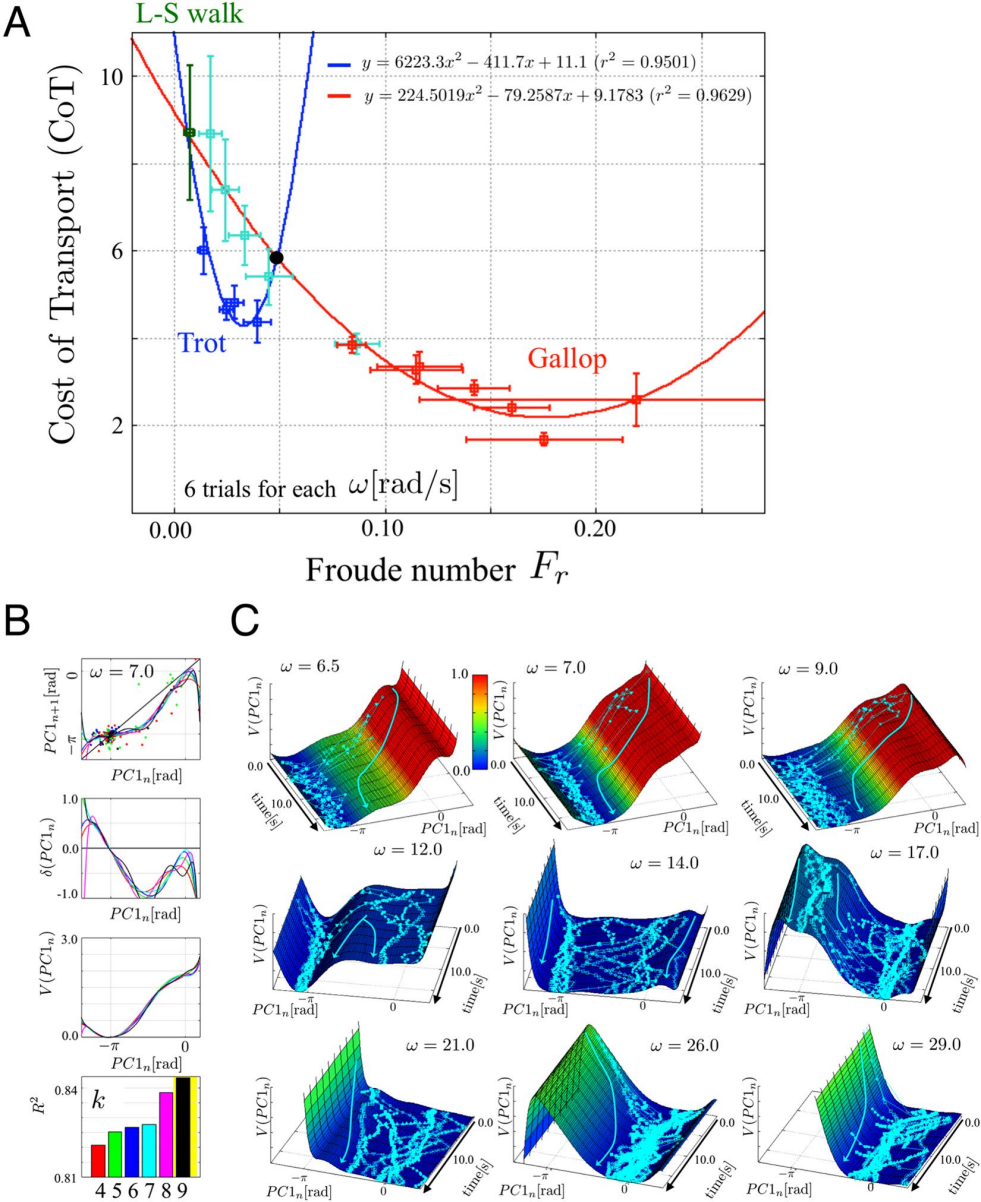
Efficiency and stability. (**A**) Cost of transport (CoT) versus Froude number *F*_*r*_ for each value of *ω*. The colours of data points represent the range of *ω* values: dark green represents a value less than 6.5, blue represents values ranging from 7.0 to 9.0, cyan represents values between 12.0, and 19.0, and red represents values between 21.0 and 29.0. The blue and red lines represent the best-fitting second-order polynomial curves according to least-squares regression analysis for *ω* < 10 (blue) and *ω* > 10 (red). (**B**) The graphs from the top represent return maps *PC*1_*n*+1_ = *f*(*PC*1_*n*_), *δ*_*PC*1_(*PC*1_*n*_), potential functions *V*_*PC*1_(*PC*1_*n*_), and *R* ^2^ values obtained using polynomial models for approximation for *ω* = 7.0 rad/s. (**C**) Convergence of *PC*1_*n*_ (order parameter) on the surfaces of the potential functions, which describes gait stability. Here, we use polynomials (*k* = 9). In this figure, the horizontal *x*, *y*, and vertical *z* axes represent *PC*1, time *t*, and the value of the potential function *V*_*PC*1_(*PC*1), respectively. The light blue trajectories represent the phase convergence for trials with different initial conditions. The time courses of phase convergence depending on initial conditions were in line with the shapes of potential functions, as shown by arrows in these graphs. In particular, bimodal structures of potential functions at *ω* = 12.0, 14.0, and 17.0 indicate two possible steady states depending on initial conditions.

We measured the energy consumption of the robot in steady gait patterns and calculated the “cost of transport (CoT)^28^” for each value of *ω*. Figure 3(A) shows the averages and standard deviations (SDs) of the CoT versus the “Froude number” *F*_*r*_ for six trials of each value of *ω*. The results indicated that the trot and gallop were the most energy-efficient gaits in the corresponding range of Froude numbers. This observation agrees with biological data in this regard^2^. Moreover, we found that, for the same Froude number range, the canter was a less efficient gait pattern than the trot (Fig. 3(A)). One possible consideration is that most quadrupeds would more frequently exhibit a trot rather than a canter in this velocity range. Another possible consideration is that our model does not include an “intralimb coordination” mechanism because of its simplicity, resulting in insufficient velocity for a canter, as discussed in the Supplementary Materials (SM).

### Gait Stability Analysis

To analyse the underlying mechanism during gait transition more intensively and to discuss gait stability quantitatively, we conducted stability analysis of steady gait patterns (phase differences between oscillators *ϕ*_*i*_) using a ‘return map’, i.e. a one-dimensional Poincaré map^29, 30^. Using principle component analysis (PCA), we extracted an ‘order parameter’^31^ (PC1 component in PCA) that clearly represents gait patterns for gait stability analysis. Furthermore, by using a technique^10^ to extract potential functions which describe gait stability, we could verify stability using experimental data as shown in the ‘Methods’ section in detail and as illustrated in Fig. 3(C). Finally, using the obtained potential functions, we visualized the phase convergence of the PC1 component (order parameter) on the surfaces of the potential functions.

Figure 3(C) shows the convergence of *PC*1 over 20 s from various initial conditions. In this figure, the horizontal *x*, *y*, and vertical *z* axes represent *PC*1, time *t*, and the value of the potential function *V*_*PC*1_(*PC*1), respectively. Here, we use polynomials for regression (*k* = 9). The light blue trajectories represent the phase convergence for trials with different initial conditions. The time evolutions of the phase convergence of *PC*1 depending on initial conditions were in line with the shapes of the potential functions, as shown by arrows in these graphs. Therefore, potential functions could capture the dynamical structure in the gait patterns generated by our quadruped robot with the *non-wired* CPG model. In particular, the bimodal structure in the potential function at *ω* = 12.0 and 14.0 indicates two possible steady states depending on the initial conditions. We explain the results of this analysis in detail in the SM.

## Discussion

This was the first study of its kind to demonstrate that spontaneous gait transition, from walking to trotting to cantering to galloping, could be achieved by changing one parameter related to speed. Since the pioneering work of Raibert^32^, the mechanical coupling of leg controllers through the body has been studied, but there have been no studies in which gaits were reproduced in a completely self-organized manner. Previous studies have proposed local load feedback^13, 33^ with models that use threshold values of loading to switch between the stance and swing phases. By exploiting this property, Ekeberg’s model^13^ reproduced interlimb coordination in the hind legs. However, the gait patterns obtained and their performances depend on the threshold values used. Indeed, in Maufroy’s model^33^, parameter modification is used for leg clearance in the fore and hind legs (Fig. 5 in the paper^33^) for the generation of a walking gait and the “ascending coordination mechanism (ACM)” (Section 4.3 in the paper^33^) is added for explicit ipsilateral leg coordination against external perturbations. To clarify and be more precise, the setting of a higher threshold value (less than approximately one quarter of the model weight) results in high sensitivity to body properties and perturbations. Furthermore, previous models did not continuously modulate the phase through loading feedback except during the switch timing of stance and swing phases; thus, the angular velocity of the phases was constant during the stance phase. In our CPG model, on the other hand, oscillator phases were modulated in real time according to the magnitude of local load information *N*_*i*_, which sufficiently reflects the physical situation of the other legs. This situation-dependent phase modulation in Eq. (1) enables leg movements to be coordinated in accordance with their locomotion speed and physical properties^20^ by fully exploiting physical communication between legs. Furthermore, the resulting observations in our model explain many aspects of quadruped locomotion and gait transition mechanisms. Some potential advantages of our model with continuous phase modulation include extended abilities, e.g. to negotiate uneven terrain or to generate versatile behaviours, e.g. turning, by adding only simple mechanisms. For hexapod walking, ‘Walknet’ and its extended models have reproduced smooth gait transitions in insects^16^, where leg coordination was achieved using neural information exchange between neighbouring legs: hence, ours is the first *radical* local sensing approach for leg coordination via physical communication in speed-dependent gait transition.

Our model also has an implicit threshold value (*ω*/*σN*_*i*_) for the transition between “excitatory” and “oscillatory” behaviour from the viewpoint of an “active rotator” model^34^. The properties transition automatically depending on the continuous value of the load-sensing information *N*_*i*_, as discussed in ref. 20. More interestingly, the properties also change depending on the parameter used for the intrinsic angular velocity *ω* of the oscillators. The excitatory property is dominant in low-speed locomotion, i.e. walking, whereas the oscillatory property is dominant in middle- and high-speed locomotion, i.e. trotting and galloping. The transition of these properties with the change in *ω* ^20^, which is generated by the balance between *ω* and *σN*_*i*_ (first and second term in Eq. (1)), also contributes to spontaneous gait transition with our CPG model through modification of the potential functions in Fig. 3(C), which describes the stability of each gait. Past biological studies have investigated gait transition in animals^31, 35–37^. Gait transition in humans^31^ and quadrupeds^10, 38, 39^ as well as left- and right-hand coordination^40^ and limb coordination between two people^41^ have been analysed from the viewpoint of “non-equilibrium phase transition” phenomena. In our model, the obtained gait patterns had a stable phase relationship and minimum energy expenditure. Moreover, gait transition occurred at the point where the system lost its stability and had to reduce its energy cost, as shown in Fig. 3. Our study is the first experimental evidence for trot-gallop gait transition that considers the non-equilibrium phase transition of nonlinear dynamical systems, including general characteristics, e.g. “hysteresis”, “critical fluctuation”, and “critical slow convergence” in transition regions (see the SM for further details).

Biological evidence that locomotion is modulated via feedback from load-sensitive receptors^42, 43^ supports our findings concerning load-dependent interlimb coordination. Furthermore, based on biological insights^42–45^, we know that animals have two types of sensory organs: “tonic” and “phasic” receptors. A tonic receptor senses continuous values of a sensory input, e.g. loading, whereas a phasic receptor senses the timing of changes in the sensory input, e.g. touchdown. Most previous studies focused mainly on the phasic receptor function^10, 13, 33^. Although the functional role of these receptors requires further detailed investigation, our model can help to explain the functional role of the tonic receptor for interlimb coordination in quadruped locomotion.

Our work in this study also provides a suggestive lesson for the field of robotics. Most traditional approaches^46, 47^ in the field are based on “centralized” approaches, in which a controller governs all “degrees of freedom” (DoFs) at all times to track the desired trajectory of each point on a robot’s body, resulting in high computational cost. Using local load sensing, we can efficiently detect information concerning the robot’s dynamic interaction with its environment. As a result, our model effectively reduces the computational resources required for leg coordination by exploiting physical communication. This may constitute the basis of an unconventional approach to coordinating the large numbers of DoFs required for robot locomotion, and could lead to a wide range of applications such as adaptive legged robots working in disaster areas, user-friendly legged entertainment robots, and automatic motion-creation algorithms for computer graphics (CG) animations.

## Methods

### Quadruped robot

**Figure 4:**
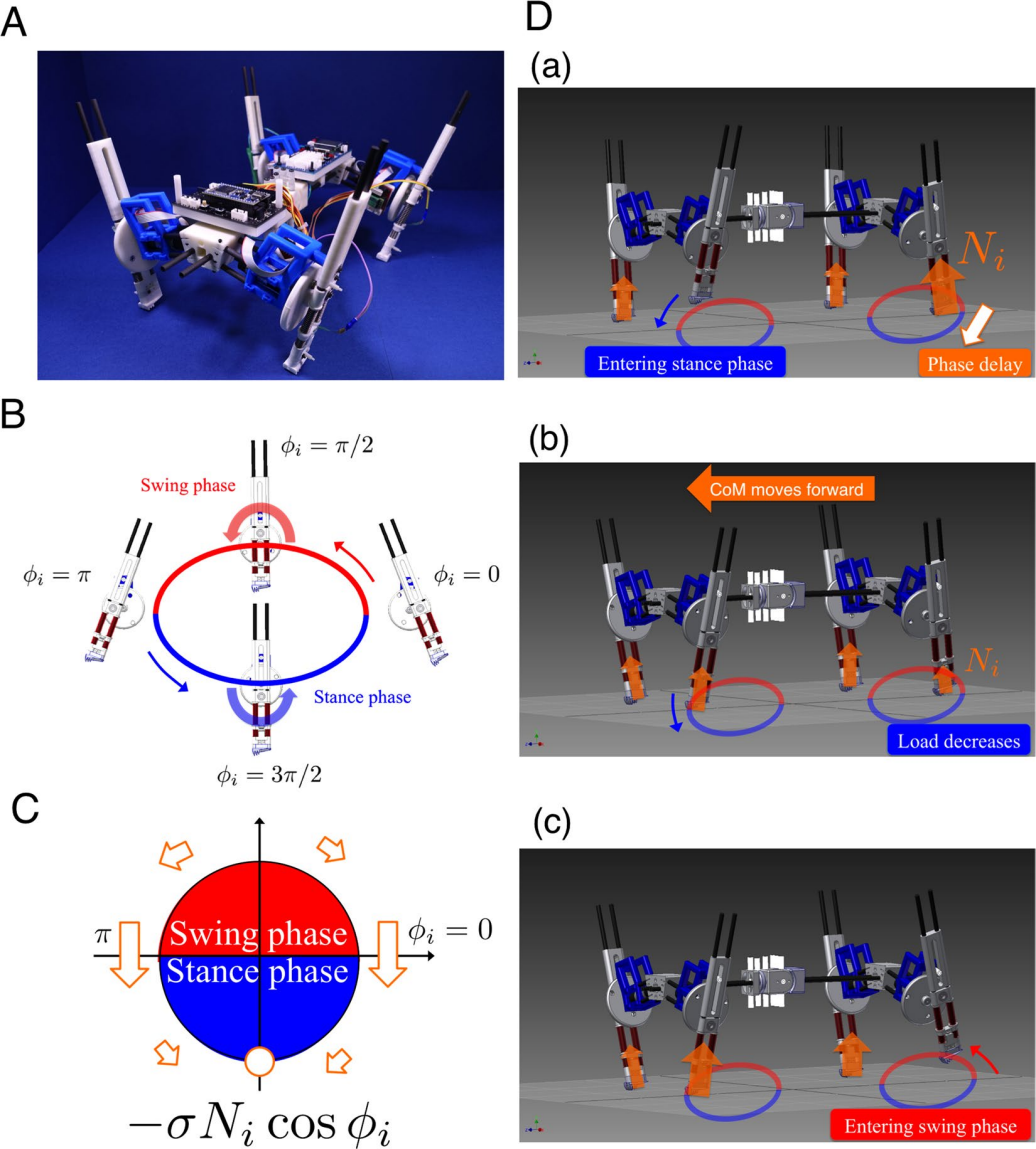
The quadruped robot, *Oscillex 3*. (**A**) The robot is 0.25 m long and 0.20 m wide and weighs 2.0 kg. (**B**) Each leg has a DC motor to generate rhythmic swing-stance leg motion through a crank mechanism. (**C**) The feedback effect on the oscillator phase. (**D**) Interlimb coordination mechanism through *physical communication*.

Our quadruped robot (Fig. 4(A)), *Oscillex 3*, is designed on the basis of the following simplifications: (i) A simple leg with one DoF is implemented, which allows us to ignore intralimb coordination. (ii) We use a “phase oscillator”^48^, which we consider to be the most abstract model that can generate rhythmic neural commands for a leg, as the basic component of our CPG model. (iii) We use neither predefined neural connections between the oscillators nor a preprogrammed phase relationship between them.

Figure 4(A) shows the structure of our quadruped robot. The robot consists of four leg segments and a backbone segment, as shown in Figs S1 and S2 in the Supplementary Materials (SM), respectively. The total weight of the robot is approximately 2.00 [kg]. The lengths of the backbone and each leg are 0.24 [m] and 0.20 [m], respectively. We used a direct-current (DC) motor (Maxon Japan Corporation: RE-max 17, MR, and GP16A) for each leg. The mechanism shown in Fig. 4(B) converts the rotational motion of the DC motor into limb motion during the swing and stance phases (Movie S7). Furthermore, we attached pressure sensors (Interlink Electronics: FSR400) to the feet of the robot to detect ground reaction forces (GRFs), as shown in Fig. S4 and Movie S8. A schematic of the whole control system for our robot is shown in Fig. S6. Each leg has a motor driver circuit as shown in Figs S1(A) and S6. The backbone contains a main control board and a power regulator board (Figs S2(A) and S6). We calculated the oscillator phase in each leg using a micro controller (mbed NXP LPC1768) on the main control board. We manipulated each DC motor on the legs using proportional-integral-derivative (PID) control so that its rotational angle corresponded to the oscillator phase. The SM describe the design characteristics of our robot and its physical parameters in detail.

### CPG model

We used our CPG model^20^ for the gait transition experiments. Our model is described by the following equation:

1 $${\dot{\varphi }}_{i}=\omega -\sigma {N}_{i}\,\cos \,{\varphi }_{i}$$

where *ϕ*_*i*_ denotes the oscillator phase, *ω* denotes the intrinsic angular velocity, and σ denotes the weight of local sensory feedback. *N*_*i*_ represents the ground reaction force (GRF), which was detected using a pressure sensor on each foot. We controlled each leg with a DC motor according to the corresponding oscillator phase to generate rhythmic leg motion in the swing and stance phases within a given period (Fig. 4(B)).

We now explain how interlimb coordination is achieved using the CPG model (Eq. 1). Based on the effect of local sensory feedback, the oscillator phase is modulated toward 3*π*/2 when *N*_*i*_ > 0 (Fig. 4(C)), which means that the corresponding leg remains in the stance phase while supporting the body. As shown in Fig. 4(D), (a) when the left hind (LH) leg supports the body at the end of its stance phase, a phase delay is introduced depending on the magnitude of *N*_*i*_. This phase delay allows time for the left fore (LF) leg to enter the stance phase and begin to support the body. (b) This effect causes the centre of mass (CoM) of the body to move forward, which in turn reduces the load on the LH leg. (c) As a result, the phase delay on the LH leg decreases, allowing the LH leg to enter the swing phase. Note that *N*_*i*_ includes information regarding the physical situation of the other legs at the given time, which enables the model to fully exploit the physical communication between leg movements without direct coupling between oscillators unlike most previous CPG models^6–11, 39^.

### Leg coordination analysis using SVD

In refs 24–26, movement decomposition was conducted by using principle component analysis (PCA) based on elevation angle space, i.e. the angles between links and vertical line, Cartesian coordinates, and multi-joint angle space, respectively. Here, we used a 4D coordinate system *R*(***θ***, *t*) = [*θ*_*LF*_(*t*), *θ*_*LH*_(*t*), *θ*_*RF*_(*t*), *θ*_*RH*_(*t*)] (rotational angles in the shoulder/hip joint for four legs) measured by a 3D motion capture system. Thus, we ultimately obtain four principle components through the analysis. The movement of gait transition can be expressed by writing the time-series data of leg angles as a column in the matrix *R*(***θ***, *t*)^23, 24^ as shown in Fig. S10 (SM). Principle component analysis is conducted by finding the eigenvectors and eigenvalues of the covariance matrix of object data, e.g. *conv*(*R*(***θ***, *t*))^25^ (or those of the correlation matrix^26^). By using SVD, the eigenvectors *z*_*i*_(***θ***) of *conv*(*R*(***θ***, *t*)) can be easily calculated as the following equation:

2 $$R({\boldsymbol{\theta }},t)=U(t){\rm{\Lambda }}{Z}^{T}({\boldsymbol{\theta }})=\sum {\lambda }_{i}{u}_{i}(t){z}_{i}({\boldsymbol{\theta }})$$

where *U*(*t*) = [*u*_1_(*t*) *u*_2_(*t*) *u*_3_(*t*) *u*_4_(*t*)], Λ = diag(*λ*_1_, *λ*_2_, *λ*_3_, *λ*_4_), *Z*(***θ***) = [*z*_1_(***θ***) *z*_2_(***θ***) *z*_3_(***θ***) *z*_4_(***θ***)]. *λ*_*i*_ *u*_*i*_(*t*) represents time series values with corresponding eigenvectors *z*_*i*_(***θ***). Thus, the time series data *R*(***θ***, *t*) of movements can be decomposed into time-invariant spatial patterns *z*_*i*_(***θ***) (four components shown in the left figure in Fig. 2(A)), and temporal characteristic patterns *λ*_*i*_ *u*_*i*_(*t*) of corresponding spatial patterns (shown in the middle figure in Fig. 2(A)) by applying SVD^23, 24^. In our case, average data (average leg angles) were almost 0, therefore, we did not subtract average data, e.g. *R*_0_, from *R*(***θ***, *t*) unlike^24–26^. The reasons for employing SVD were as follows: (1) The internal sensory data regarding the *N*_*i*_ of our robot was slightly too noisy to permit continuous quantitative analysis of the gait transition. (2) It was important to analyse experimental results using *objective* and *external* data, e.g. using a 3D motion capture system, to confirm the accuracy of our experiments. (3) SVD analysis can divide time-series data into spatial and temporal patterns, e.g. using MATLAB functions, and therefore permits visualization of the contribution ratios of these spatial and temporal patterns to the corresponding gait at any given time.

Figure 2(A–C) (Fig. S13 in the SM, the total period in the gait transition experiment) show the patterns underlying the quadruped gait transition experiments—spatial patterns *z*_*i*_(***θ***) (at left in (A)), temporal patterns *λ*_*i*_ *u*_*i*_(*t*) (at middle in (A) and at left in (B), (C)), and leg angle data *R*(***θ***, *t*) (at right in (A–C)). These are arranged in descending order of the weights of their singular values *λ*_*i*_, i.e. the contribution ratios of the patterns (shown to the far left of Fig. S13(A) from top to bottom). As shown in Fig. 2(A) left, from the top graph, the spatial patterns correspond to *trotting* (blue), *pronking* (green), *bounding* (red), and *pacing* (cyan), in which each foot is coloured when the spatial pattern value *z*_*i*_(***θ***) of the corresponding leg is negative. The spatial patterns are time-invariant; therefore, they are common for (A–C) during the gait transition experiment.

### Measuring energy efficiency and locomotion velocity

To calculate the energy efficiency in the gait patterns obtained, we measured the consumption current *I*(*t*) [A] using a current sensor (SparkFun: ACS712) over a period *T* [s] during the steady gaits for each value of *ω* [rad/s]. We also measured the distance traveled *D* [m] on the treadmill during the same period. Using these data items, the “cost of transport”^28^ (CoT) was calculated from the following equation:

3 $$\mathrm{CoT}\,=\frac{{\int }_{0}^{T}P(t)dt}{mgD}$$

where *P*(*t*) = *I*(*t*) × *V*(*t*) [W] represents the energy consumption (here, *V*(*t*) was 12 V) of the four leg motors during locomotion, *m* [kg] represents the total mass of the robot, and *g* [N/m ^2^] is the gravitational acceleration. The locomotion velocity was also calculated using *v* = *D*/*T* [m/s]. We calculated the non-dimensional Froude number *F*_*r*_^49^, i.e. the scale-independent locomotion speed as follows:

4 $${F}_{r}=\frac{{v}^{2}}{gl}$$

where *l* [m] represents the length of the leg of the quadruped robot.

### Stability Analysis Using a Return Map

We used the following procedure for the gait analysis:

1. Dimensional reduction for gait representation (phase differences between oscillators *ϕ*_*i*_) using PCA to extract an “order parameter”^31^ (see details in the SM).
2. Gait stability analysis using a regression model for the approximation of return map, and
3. Visualization of the potential functions that describe gait stability.

We conducted a stability analysis of the obtained steady gait patterns (order parameter) by using a “return map”, i.e. a one-dimensional Poincaré map^29, 30^. The top panels in Fig. 3(B) (Fig. S19 in the SM) show the return maps of the representative *ω* = 7.0 red/s for *PC*1 (order parameter). The horizontal and vertical axes represent *PC*1 at the (*n*)th and (*n* + 1)th steps, respectively. The intersection between the functions *PC*1_*n*+1_ = *f*(*PC*1_*n*_) and *PC*1_*n*+1_ = *PC*1_*n*_ (bold black line) in the graph shows fixed points, i.e. periodic solutions (stable or unstable gait patterns). If the slope of the return map around these points is less than unity, then the solution is stable; otherwise, the solution is unstable.

We selected regression models that can approximately formulate return maps *f*(*PC*1_*n*_) for gait analysis. Here, we selected functions as regression models as follows:

5 $$PC{1}_{n+1}=f(PC{1}_{n})=\sum _{i=1}^{k+1}{a}_{i}{(PC{1}_{n})}^{k+1-i}\mathrm{.}$$

By using these functions, we estimated potential functions to visualize and discuss the dynamical structure of the target system^10^. Here, we define *δ*_*PC*1_ (*PC*1_*n*_) as follows:

6 $${\delta }_{PC1}(PC{1}_{n})=PC{1}_{n+1}-PC{1}_{n}$$

7 $$=f(PC{1}_{n})-P{C}_{n}\mathrm{.}$$

By integrating −*δ*_*PC*1_(*PC*1_*n*_) from −1.5*π* to *PC*1_*n*_(≤0.5*π*), we obtain the following function *v*_*PC*1_(*PC*1_*n*_):

8 $${v}_{PC1}(PC{1}_{n})={\int }_{-1.5\pi }^{PC{1}_{n}}-{\delta }_{PC1}(PC{1}_{n})dPC{1}_{n}\mathrm{.}$$

We define the potential function *V*_*PC*1_(*PC*1_*n*_) by using ${v}_{PC{1}_{n}}$ as follows:

9 $${V}_{PC1}(PC{1}_{n})={v}_{PC1}(PC{1}_{n})-{{\rm{\min }}}_{PC1\in [-1.5\pi ,0.5\pi ]}{v}_{PC1}(PC{1}_{n})$$

10 $$({V}_{PC1} > 0\,{\rm{for}}\,PC{1}_{n}\ne {\rm{\arg }}\,{{\rm{\min }}}_{PC1\in [-1.5\pi ,0.5\pi ]}{v}_{PC1}(PC{1}_{n}).$$

By using the potential function, we can verify the gait stability.

The two middle panels in Fig. 3(B) show the functions *δ*_*PC*1_(*PC*1_*n*_) and *V*_*PC*1_(*PC*1_*n*_) for representative *ω* = 7.0. The colours of the lines denote the differences in *k*, which is the degree of the polynomial models. These results (all of which were verified to have *R* ^2^ in the bottom panels of more than 0.75, see Fig. S20 in SM) indicate that the degree of regression did not significantly affect the number and location of the periodic stable/unstable solution (Fig. 3(B)). The stable solutions obtained by using return maps (convergent states) are consistent with the data plot of phase convergence in Fig. S18 in the SM. Therefore, we concluded that the return maps and potential functions are sufficient for analysing gait stability as in the previous works^10, 29, 30^.

## Electronic supplementary material

Supplementary Materials
