# Supplementary material for: A Quadruped Robot Exhibiting Spontaneous Gait Transitions from Walking to Trotting to Galloping
Source: Sci Rep. 2017 Mar 21;7:277. doi: 10.1038/s41598-017-00348-9 (PMC5428244; doi:10.1038/s41598-017-00348-9)
Supplement: Supplementary file 1 — Supplementary Materials [file 41598_2017_348_MOESM1_ESM.pdf]

# Supplementary Materials for

## A Quadruped Robot Exhibiting Spontaneous Gait Transitions from Walking to Trotting to Galloping

Dai Owaki, Akio Ishiguro

Corresponding author: owaki@riec.tohoku.ac.jp

**This PDF file includes the following:**

Materials and Methods
References
Table S1
Figs. S1 to S23

All photograph in the all figures are taken by authors.

**Other Supplementary Materials for this manuscript include the following:**

Movies S1 to S10
Download URL:
https://fsa.fir.riec.tohoku.ac.jp/fircloud/index.php/s/Dj3dURHM27Yip13

The person who conducted gait transition experiment (Movie S1) was the first author (Dai Owaki), who grants full permission for the image to be used in publication online. If you cannot download the above file, please contact us.

## Materials and Methods

In the main text, we present our load-dependent interlimb coordination model, which enables spontaneous quadruped gait transition. Here, we provide reviews of the state-of-the-art on legged locomotion, hardware implementation of our model, and the experimental conditions of this study, as well as our methods for measuring and analyzing data.

In Section 1, we describe contemporary research on interlimb coordination in legged locomotion. Section 2 is dedicated to a description of the design characteristics of our quadruped robot and its physical parameters. In Section 3, we describe methods for measuring three-dimensional (3-D) kinematic data regarding the robot's locomotion. In Section 4, we describe the measurement methods for some gait parameters. In Sections 5 and 6, we describe the analysis of the average duty factor during the gait transition experiment and the stability analysis of gait patterns using a "return map", i.e. a one-dimensional Poincaré map. In Section 7, we show the reproducibility and reversibility of gait transitions in our robot. Finally, in Section 8, we discuss the limitations of our model by comparing it with biological insights.

## 1. State of the art

Table S1 summarizes representative studies on this topic conducted over the last 25 years. We summarize some important work below.

(*32*):
Overview: The general goal was to elucidate quadruped running as a problem in dynamic balance and control and the specific goal was to apply a control algorithm for one- and two-legged running robots to four-legged running. The robot used a control method with a "virtual leg" (*50*), in which a pair of legs (diagonal legs for trotting, lateral legs for pacing, and fore/hind legs for bouncing) is controlled such that leg movements are almost identical within a pair.
Review: This is Raibert's pioneering work regarding quadruped bouncing. He focused on mechanical coupling between leg controllers throughout the body. However, if his robot changes gait, the pair of virtual legs should be changed to another pair for another gait, implying that the gait patterns were not generated in a self-organized manner.

(*6*):
Overview: The specific goal was to investigate whether a "hard-wired" central pattern generator (CPG) can produce gait patterns in quadrupeds and to investigate the model independence of the CPG model. The authors reproduced gait transitions from walking to trotting to bouncing using the proposed hard-wired CPG model.
Review: This treats gait transition by changing more than four parameters in each model (*Stein CPG*, *Van der Pol* model, and *FitzHugh-Nagumo* model). No embodiment is considered (the body and the interaction between the body and the environment were not modeled).

(*15*) and (*16*):
Overview: The goal was to develop a system as a scientific tool to investigate insect walking. The authors proposed a sensory-driven leg coordination model, called

*WalkNet,* for hexapod locomotion. This model reproduced behaviors (e.g., walking, turning, etc.) in stick insects.
Review: This (*16*) also treats gait transition between tetrapod and tripod. The model use neural informational exchange among neighboring legs for leg coordination.

(*51*) and (*52*):
Overview: The goal was to understand the control mechanisms necessary to maintain their speed, height, and postural orientation in a quadruped's trotting and galloping gaits. The authors proposed a horse-like model that can reproduce trotting (*51*) and galloping (*52*) gaits by incorporating the body and its interactions with the environment. Their models are based on the biomechanics of a horse's locomotion. The model had neck and back joints in the backbone.
Review: This work involved a two-dimensional (2D) model and simulations only. Trot and gallop gait patterns were generated through different gait generator models based on kinematic data. Thus, the gait patterns were not generated in a self-organized manner.

(*53*):
Overview: The goal was to introduce a framework for representing, generating, and tuning the gaits of a hexapod robot. A touchdown-modulated clock coordination mechanism was proposed for a hexapod robot. This is simple and easy to implement in a robot using a hip joint sensor to detect touchdown in the robot.
Review: The authors discussed neither adaptability to changing body properties nor gait transition.

(*13*):
Overview: The goal was to examine some of the sensory mechanisms involved in controlling the stepping of the hind legs of walking cats using computer simulations. The authors showed that a reflex mechanism for unloading is necessary for the generation of locomotion without inter-leg neural communication using a simulation model for a cat with only hind legs.
Review: This work showed that a neuromuscular simulator can be used to explain a biological mechanism. However, there is a lack of adaptability to changing body properties and no discussion of the gait transition owing to the use of a threshold value for switching between stance and swing phases.

(*54*):
Overview: The goal was to theoretically elucidate why a horse prefers a canter or gallop to a pronk using a simple model with the collisions upon the touchdown of legs. The model explains that the three-beat canter is more efficient than the one-beat pronk. The authors concluded that this is why horses choose to canter during high-speed locomotion.
Review: This work is too simple to explain the generation mechanism of gait patterns. This model cannot explain the footfall sequence in gaits and the work involves no embodiment.

(*33*):
<u>Overview</u>: The general goal was to integrate posture control and rhythmic motion control in the framework of a limit-cycle-based control architecture. The authors showed that a loading/unloading local feedback rule generated adaptive walking in a 3D simulation, including whole-body dynamics.
<u>Review</u>: There is a lack of adaptability to changing body properties and no discussion of the gait transition owing to the use of a threshold value for switching between stance and swing phases. The work necessitates strictly tuning the body and control parameters for the generation of various gait patterns and robustness against perturbations.

(*10*):
<u>Overview</u>: The goal was to clarify the hysteresis mechanism in the walk-trot transition of quadrupeds. The authors proposed a hard-wired CPG model with sensory feedback for touchdown (phase resetting). They explained the mechanism of hysteresis in the walk-trot gait transition using stability analysis based on the return maps and potential functions.
<u>Review</u>: This work considered only the walk-trot transition. Touchdown modulation (phase resetting) could not reproduce adaptability to changes in body properties, as in Ekeberg's method (*13*) for loading.

(*11*):
<u>Overview</u>: The goal was to reveal the intrinsic principle of gait generation in quadrupeds. The authors proposed a hard-wired CPG model with sensory feedback for loading and posture control of the body. The model reproduced various gait patterns depending on the posture of the body.
<u>Review</u>: This work involves plantar simulation. No verification of the robotic platform was done.

## 2. Design characteristics of the quadruped robot

### Detailed structure of the quadruped robot

Figures S1 and S2 show the detailed structures of a leg and the backbone, respectively. The leg and backbone consist of carbon fiber rods and acrylonitrile butadiene styrene (ABS) resin printed using a 3-D printer because we wanted to maintain a balance between the robot's weight and its strength. As shown in the profile view (Fig. S3(A)), the legs are attached perpendicular to the backbone segment. To ensure stable body motion along the roll axis and effective swing and stance leg motion, we attached each leg to the backbone segment at an inclination of 15 [deg] from a line perpendicular to the ground from the frontal view (Fig. S3(B)). We implemented passive springs (MISUMI: UF8-30) in each leg for shock absorption and to allow the leg to push off from the ground (Fig. S4). In the backbone, we incorporated silicon tubes to implement a spring-like mechanism along the pitch and roll axes, as shown in Figs. S5(A) and (B). By adjusting the number of silicon tubes, we were able to adjust the stiffness of the backbone (Fig. S7). These mechanisms allow the backbone to deform according to the terrain, leading to the generation of physical communication among the legs (Movie S9).

### Physical parameters of the quadruped robot

Here, we describe the parameters that contribute to the gait transition of our quadruped robot.

Elasticity of the springs in the backbone and legs

We measured the stiffness, i.e. the angle–torque relationship, of the pitch and roll joints in the backbone (Fig. S5). Figure S7 shows the measured stiffness of each joint. We measured four combinations of the applied load [N] and its distance [mm] from the center of joint rotation for six displacement angles [rad]. The data show that, (i) the pitch joint stiffness has an asymmetric profile upward and downward, whereas the roll joint has a symmetric profile; and (ii) the approximate linear stiffness of the pitch joint (3.02 Nm/rad) was three times greater than that of the roll joint (1.06 Nm/rad). This difference would contribute to backbone oscillation in the trot and gallop (Fig. S12), resulting in spontaneous gait transition based on *physical communication*. The stiffness of the leg springs (MISUMI: UF8-30) was 0.49 N/m.

Position of the CoM

We measured the center of mass (CoM) position using a measurement system for vertical ground reaction forces using a pressure sensor sheet (NITTA, BIG-MAT). Fig. S8 shows the top view of our robot on the sensor sheet. The CoM position was located close to the center of the body.

Geometric toe trajectory

The geometric path of the toe affects gait stability (*57*). For our robot, we chose the parameter values (Fig. S9) for the path by tuning them through trial and error to achieve sufficient foot clearance and step size for the gait transition. Using the parameter values $a$ = 0.040 m, $r$ = 0.015 m, and $l$ = 0.080 m, we obtained foot

clearances $r_u$ = 0.00991 m and $r_l$ = 0.02009 m and step size of 2 $r_s$ = 0.08618 m. Note that the *actual* ground clearance and stride length are determined via the interaction between the robot and its environment, as shown in Fig. S14(D).

Relative dimensions of the legs versus body length and width

We show the body length (backbone length) as $b_l$ = 0.24 m and width $b_w$ ≒ 0.17 m (Fig. S3). For a leg length $l$ = 0.080 m, the dimensions of the legs relative to the body length and width are $b_l/l$ = 3.00 and $b_w/l$ = 2.13.

**Experimental conditions**

We conducted our gait transition experiment on a treadmill, as shown in Fig. S10(A). The experimenter gradually changed the speed of the treadmill by hand tuning according to the intrinsic angular velocity $\omega$ of the oscillators such that no inertial force was applied to the robot. Thus, the speed of the treadmill had almost no effect on the gait patterns obtained. The low friction (static and dynamic friction) on our treadmill led to a looser grip of the foot on the treadmill, resulting in a lower Froude number in Fig. 3 (A) and Fig. S14(B) than in an animal's trot and gallop. To improve the Froude number, we can employ various approaches, e.g. replacing the ground conditions of the treadmill or changing the sole properties of the quadruped robot's foot to increase the grip force.

**Adaptability to changes in body properties**

We describe here the robustness of our model against changes in body properties (Fig. S11). As shown in a previous study (*20*), one of our quadruped robots, *Oscillex 1,* exhibited L-S and D-S walks depending on the position of the CoM. Our robot exhibited an L-S walk with a load on the fore legs and a D-S walk with a load on the hind legs. We also showed that another of our quadruped robots, *Oscillex with a pendulum*, exhibited exclusively pace and trot gaits, respectively, in cases with and without a physical pendulum attached to it (*56*). This was because the *continuous* value $N_i$ included essential information regarding the physical situation of the other legs at any given time. This approach was completely different from previous approaches (*13, 33*) that involved the use of a threshold value for loading in order to switch between the stance and swing phases.

## 3. Kinematic measurements and leg coordination analysis of gait transition

### Kinematic measurements of robot locomotion

Robot locomotion was recorded using a real-time motion capture system with six high-speed cameras (OptiTrack Prime 17W) at 360 frames per second. The procedure was as follows: (1) twenty-four markers were attached to the robot (four on the fore backbone, four on the hind backbone, four on each leg) to measure real-time 3-D position data (Fig. S10(B)). (2) Using the position data from the markers, we calculated six angles ($\theta_{roll}$, $\theta_{pitch}$, $\theta_{LF}$, $\theta_{LH}$, $\theta_{RF}$, and $\theta_{RH}$) along the roll and pitch joints of the backbone and each leg joint, as shown in Fig. S10(C).

Figures S12(A) and S12(B) show the representative profiles of the six angles of the kinematic data ($\theta_{roll}$, $\theta_{pitch}$, $\theta_{LF}$, $\theta_{LH}$, $\theta_{RF}$, and $\theta_{RH}$) in the gait transition experiment illustrated in Fig. 2 (A) in the manuscript file and Movie S1: (a) an L-S walk, (b) a trot, and (c) a gallop. These data are plotted on the corresponding gait diagrams. As shown in Fig. S12(A), in the trot and the gallop, roll and pitch oscillations, respectively, were observed in the backbone. In the walk, the synchronization of the pitch and roll oscillations could generate yaw-like oscillation similar to that which occurs in quadruped animals (*63, 64*) because of the lack of a yaw joint in the backbone. These backbone oscillations might contribute to physical communication-based interlimb coordination in quadruped gait transition. Moreover, in the locomotion of actual quadrupeds, head movements affect the yaw spine movements for walking and the pitch spine movements for galloping gaits (*63, 64*).

Figure S12(B) illustrates the correlation between leg joint angles and gait diagrams based on pressure sensor values. As this figure shows, the foot contact periods correspond to periods of decrease in the joint angles except in the case of the gallop. In the gallop, the delay in touchdown in periods during which the joint angles decrease corresponds to the decrease in the foot stroke length. Figure S12(C) illustrates the correlation between oscillator phases and gait diagrams. This graph indicates that the relation $\phi_i = \pi$ nearly corresponds to the timing of touchdown.

The movement of gait transition can be expressed by writing the time-series data of leg angles as a column by the following matrix (*23, 24*) as shown in Fig S10:

$$R(\boldsymbol{\theta}, t) = \overset{\text{Leg angles}\ \longrightarrow}{\begin{bmatrix} \theta_{LF}(t_0) & \theta_{LH}(t_0) & \theta_{RF}(t_0) & \theta_{RH}(t_0) \\ \theta_{LF}(t_1) & \theta_{LH}(t_1) & \theta_{RF}(t_1) & \theta_{RH}(t_1) \\ \vdots & \vdots & \vdots & \vdots \end{bmatrix}} \Big\downarrow \text{Time}$$

Using the angle data *R(θ, t)*, we conducted leg coordination analysis through "singular value decomposition" (SVD), as shown in the main text and Fig. S13:

**4. Measuring energy efficiency, locomotion velocity, stride frequency, and stride length**

Figures S14 (A) and (B) show the cost of transport and the Froude number for each value of $\omega$ [rad/s]. We measured these values for six trials of each $\omega$ [rad/s]. The points and error bars represent the averages and standard deviations (SDs), respectively, of the results of the trials. The colors of data points represent the range of $\omega$: dark green represents a value less than 6.5, blue represents values ranging from 7.0 to 9.0, cyan represents values between 12.0 and 19.0, red represents values between 21.0 and 29.0. The blue and red lines represent the quadratic regression curves obtained using the least-squares method for $\omega < 10$ (blue) and $\omega > 10$ (red). It should be noted that the Froude number in the gray region decreased at one point as $\omega$ increased. In this region, we found non-convergent gait patterns for $\omega = 12.0$ (Movie S7) and 14.0 and a half-bound for $\omega = 17.0$ (Movie S5) and 19.0, and a canter for $\omega = 21.0$ (Movie S6). Therefore, we abruptly changed the value of $\omega$ from 9.0 to 17.0 to generate a smooth gait transition to a gallop by avoiding the unstable gap between the trot and gallop gaits. This profile was similar to a "catastrophic structure" (*59, 60*) from the viewpoint of dynamic systems, which can be seen in the results of the stability analysis described in Section 6.

To estimate stride frequency, we calculate the estimated stride period $T_{st}$ using the timings when the corresponding oscillator phase is $\phi_i = \pi$ based on the correlation between oscillator phases and gait diagrams shown in Fig. S12. We calculated the stride frequency $\omega_{st}$ [rad] from the following equation:

$$\omega_{st} = \frac{2\pi}{T_{st}} \quad \dots \text{(S1)}$$

The stride length was estimated using the following equation:

$$r_{st} = v \times T_{st} \quad \dots \text{(S2)}$$

Figures S14 (C) and (D) show the stride frequency and stride length for each value of $\omega$. The stride frequencies were slightly less than $\omega$ because of the interaction between the robot and its environment during the stance phase, in which the angular velocity of the oscillator decreases depending on the feedback term and the interaction with the ground. In the trot, the maximum step size is approximately a two-steps size $4r_s$ around the foot trajectory. In contrast, in the canter (or non-convergent region) and gallop, the stride length is approximately one step size $2r_s$ because of the low friction (static and dynamic friction) on our treadmill, resulting in a looser grip of the foot on the treadmill.

## 5. Duty factor analysis

To distinguish quantitatively between walking and running, we calculated the average duty factor during the gait transition experiment. Figure S15(A) shows the average duty factor for intervals in *T* seconds using the sensor value $N_i$. We measured the durations of the periods within which the sensor value was greater than the threshold value (10% of the maximum $N_i$, similar to visualization of the gait diagram in Fig.2 (C) of the main text), meaning that $S_{LF}$ = 1 during $T$ (=$2\pi/\omega$ ($\omega$ = 6.0) ≈1.05 s), as shown in Fig. S15(B).

In Fig. S15(A), the red and blue lines represent the average duty factors over the intervals for the fore and hind legs. The green line represents the average duty factor for all legs. During walking and trotting, the average duty factors were approximately 0.675 and 0.652, respectively, whereas the average duty factor during galloping was less than 0.5 (0.496), which implies a running gait including flight phases.

## 6. Stability analysis of gait patterns

To analyse the underlying mechanism during gait transition more intensively and to discuss gait stability quantitatively, we conducted a stability analysis of steady gait patterns using a "return map", i.e. a one-dimensional Poincaré map (*29, 30*). To analyze the stability of the target system, we must use a Poincaré map that includes its full-dimensional state variables in the mechanical and control systems of the corresponding robot. However, it is difficult to measure all of these state variables in experiments with the robot. In contrast, quadruped gait patterns can be approximately described using the phase differences $\Delta_{24}= \phi_2-\phi_4$ and $\Delta_{34}= \phi_3-\phi_4$ at the touchdown of the left fore (LF) leg, i.e. $\phi_1 = \pi$, during each locomotion period (as its Poincaré section). Furthermore, using "principle component analysis" (PCA), we extracted an "order parameter" (*31*) that clearly represents gait patterns for gait stability analysis.

Thus, we used the following procedure for gait analysis:

1. Dimensional reduction for gait representation using PCA to extract an "order parameter" (*31*).
2. Gait stability analysis using a correct regression model for the approximation of the return map.
3. Visualization of a potential function that describes gait stability.

### Convergence to gait patterns

At first, we measured the convergence of phase differences $\Delta_{24}= \phi_2-\phi_4$ and $\Delta_{34}= \phi_3-\phi_4$ at the touchdown of the LF leg, i.e. $\phi_1 = \pi$, for each $\omega$. We used four initial conditions:

trot pattern ($\phi_1,\phi_2,\phi_3,\phi_4$)=($1.5\pi$, $0.5\pi$, $0.5\pi$, $1.5\pi$);

pace pattern ($\phi_1,\phi_2,\phi_3,\phi_4$)=($1.5\pi$, $1.5\pi$, $0.5\pi$, $0.5\pi$);

bound pattern ($\phi_1,\phi_2,\phi_3,\phi_4$)=($1.5\pi$, $0.5\pi$, $1.5\pi$, $0.5\pi$); and

pronk pattern ($\phi_1$,$\phi_2$,$\phi_3$,$\phi_4$)=(1.5$\pi$, *1.5$\pi$, 1.5$\pi$, 1.5$\pi$*).

Figures S16 (A) and (B) show the convergence of gaits to a steady state (20 s) for $\omega$= 6.5, 7.0, 9.0, 12.0, 14.0, 17.0, 21.0, 26.0, 29.0 [rad/s] through more than 10 trials for each $\omega$. The different markers in these figures represent the different trials (trot, pace, bound, and pronk initial conditions).

For $\omega$=6.5, 7.0, and 9.0, the gait patterns rapidly converged to steady gaits. For $\omega$=6.5, the phase difference of $\Delta_{34}$ for most initial conditions converged to less than $\pi$, which indicates that the gait converged to a gait-like L-S walk (not a true L-S walk because of *symmetric* mass distribution in fore and hind legs (*20*)), whereas for $\omega$=7.0 and 9.0, each gait converged to a trot. For $\omega$=12, the gait patterns once converged to a trot; however, in some cases, the gait gradually destabilized. For $\omega$=14, too, the gait patterns converged once to a trot; however, they did not stabilize and converged to another pattern. These results for $\omega$=12 and 14 indicate the bistable state, where there are two stable solutions. For $\omega$=17, the gait patterns converged to a gait such as the "half-bound" gait. For $\omega$ =21, the gait patterns converged to a canter-like gait. Finally, for $\omega$=26 and 29, each gait pattern converged to a gallop.

**Dimensional reduction for gait representation (preprocessing for analysis)**

Quadruped gait patterns could be approximately described using three oscillator phases $\phi_2,\phi_3,\phi_4$ at the touchdown of the LF leg ($\sim\phi_1=\pi$ based on the results of Fig. 12 A) in each locomotion period as its Poincaré section. This three-dimensional discrete system could be reduced to a two-dimensional map by using a phase difference such as $(\Delta_{24}, \Delta_{34})_n = (\phi_2-\phi_4,\ \phi_3-\phi_4)_n$. Figure S17 (A) shows the phase plots in steady-gait patterns (during the period of 10-20 s) on a two-dimensional plane $\Delta_{24}$- $\Delta_{34}$ for various values of $\omega$. Different coloured markers represent the differences in the parameter $\omega$.

First, using "angle statistics", we can calculate the *average* angle $(\tilde{\Delta}_{24},\tilde{\Delta}_{34})$ for these data as follows:

$$(R_{i4}\cos\tilde{\Delta}_{i4}, R_{i4}\sin\tilde{\Delta}_{i4}) = \frac{1}{N}\left(\sum_{j}^{N}\cos\Delta_{j4}, \sum_{j}^{N}\sin\Delta_{j4}\right) \quad \ldots(S3)$$

where $N$ denotes the number of data (step number $\times$ the number of $\omega$ (=9)), and $R_{i4}$ denotes the length of the average vector of the phases $\Delta_{24}$ and $\Delta_{34}$.

Second, using average angles $\tilde{\Delta}_{i4}$ and the periodicity of phase data, which can transform the range of angles to $\Delta_{24} = [-1.5\pi, 0.5\pi]$ and $\Delta_{34} = [-1.0\pi, 1.0\pi]$, we re-plotted these data on a 2D plane $\hat{\Delta}_{24}-\hat{\Delta}_{34}$, where $\hat{\Delta}_{i4}=\Delta_{i4}-\tilde{\Delta}_{i4}$, as shown in Fig. S17 (B). Third, using "principle component analysis" (PCA), we extracted the principle components for gait patterns on $\hat{\Delta}_{24}-\hat{\Delta}_{34}$:

$$PC = \begin{bmatrix} 0.9890 & 0.1477 \\ -0.1477 & 0.9890 \end{bmatrix} \ldots(S4)$$

Figure S17 (C) shows the first and second components (*PC1* and *PC2*) in the $\hat{\Delta}_{24}-\hat{\Delta}_{34}$ plane. Here, *PC1* covers approximately 84% ($(\lambda_1)^2/\{(\lambda_1)^2+(\lambda_2)^2\}$ =0.8417) of the variance of all data ($\lambda_i$ denote the contribution rate of the *i*th component). This means that we can use *PC1* as a representative variable to describe gait patterns (an "order parameter" (*31*)). Using eigen vector $\boldsymbol{v}_{PC1}$ of *PC1*, as shown in Fig. S17 (C), the PC1 component is described by the following equation:

$$PC1 = \boldsymbol{v}_{PC1} \cdot (\tilde{\Delta}_{24}, \tilde{\Delta}_{34}) \ldots \text{(S5)},$$

where $\boldsymbol{v}_{PC1} = [0.9890, -0.1477]^T$. Hereafter, we use *PC1* as a one-dimensional order parameter that sufficiently describes various gait patterns.

## Convergence of PC1 to a steady state

Figure S18 shows the convergence of the PC1 variable to a steady state (at around 20 s) for $\omega$= 6.5, 7.0, 9.0, 12.0, 14.0, 17.0, 21.0, 26.0, and 29.0 [rad/s]. The different markers in these figures represent different trials with various initial conditions in more than 10 trials for each $\omega$. These results indicate that most patterns converge to one or two steady states that are distinct for each $\omega$. Interestingly, for $\omega$ =12.0 and 14.0, the gait pattern converges to two different patterns depending on initial conditions. Furthermore, "critical fluctuations" and "slow convergence" to a steady state, which are general characteristics in "non-equilibrium phase transitions" (*31*), are evident in the transition region $\omega$ = 9.0, 17.0, and 21.0.

## Gait stability analysis using a regression model

We conducted a stability analysis of the obtained steady gait patterns by using a "return map", i.e. a one-dimensional Poincaré map (*29, 30*). The top panels in Fig. S19 show the return maps of the representative $\omega$ (7.0, 17.0, and 26.0) for PC1. The horizontal and vertical axes represent *PC1* at the ($n$)$^{th}$ and ($n+1$)$^{th}$ steps, respectively. The intersection between the functions *PC1(n+1) = f(PC1(n))* and *PC1 (n+1) = PC1(n)* (bold black line) in the graph shows fixed points, i.e. periodic solutions (stable or unstable gait patterns). If the slope of the return map around these points is less than unity, the solution is stable; otherwise, the solution is unstable.

We selected regression models that can approximately formulate return maps for gait analysis. Here, we selected functions as regression models as follows:

$$PC1_{n+1} = f(PC1_n) = \sum_1^{k+1} a_i \, (PC1_n)^{k+1-i} \ldots \text{(S6)}$$

By using these functions, we estimated potential functions to visualize and discuss the dynamical structure of the target system (*10*). Here, we defined $\delta_{PC1}$ ($PC1_n$) as follows:

$$\begin{aligned} \delta_{PC1}(PC1_n) &= PC1_{n+1} - PC1_n \\ &= f(PC1_n) - PC1_n \ldots \text{(S7)} \end{aligned}$$

By integrating $-\delta_{PC1}(PC1_n)$ from $-1.5\pi$ to $PC1(\leq 0.5\pi)$, we obtain the following function $v_{PC1}(PC1_n)$:

$$v_{PC1}\left(PC1_n\right) = \int_{-1.5\pi}^{PC1_n} -\delta_{PC1}\left(PC1_n\right) dPC1_n \ldots \text{(S8)}$$

We define the potential function $V_{PC1}(PC1_n)$ by using $v_{PC1}(PC1_n)$ as follows:

$$V_{PC1}(PC1_n) = v_{PC1}(PC1_n) - \min_{PC1\in[-1.5\pi,0.5\pi]} v_{PC1}(PC1_n)$$
$$(V_{PC1} > 0 \text{ for } PC1_n \neq \arg\min_{PC1\in[-1.5\pi,0.5\pi]} v_{PC1}(PC1_n). \quad \ldots \text{(S9)}$$

By using the potential function, we can verify the stability according to the following condition:

$$\begin{aligned}\delta V_{PC1}(PC1_n) &= V_{PC1}(PC1_{n+1}) - V_{PC1}(PC1_n) \\ &= V_{PC1}(PC1_n + \delta_{PC1}(PC1_n)) - V_{PC1}(PC1_n) \\ &\sim \frac{\partial V_{PC1}(PC1_n)}{\partial PC1_n}\delta_{PC1}(PC1_n) \\ &= -\{\delta_{PC1}(PC1_n)\}^2 \leq 0 \end{aligned} \quad \ldots \text{(S10)}$$

$V_{PC1}(PC1_n) = 0$ is satisfied only for $\delta_{PC1}(PC1_n) = 0$.

The two middle panels in Fig. S19 show the functions $\delta_{PC1}(PC1_n)$ and $V_{PC1}(PC1_n)$ for representative $\omega$ 7.0, 17.0 and 26.0. The colours of the lines denote the differences in *n*, which is the degree of the polynomial models. These results (all of which were verified to have $R^2$ exceeding 0.75 in the bottom panels) indicate that the degree of regression did not significantly affect the number and location of the periodic stable/unstable solution (Fig. S19). The stable solutions obtained by using return maps (convergent states) are consistent with the data plotted for the phase convergence in Fig. S18. Therefore, we concluded that the return maps and potential functions are sufficient for analyzing gait stability as in the previous works (10, *29, 30*).

Figure S20 represents the estimated potential functions for $\omega$ 6.5, 7.0, 9.0, 12.0, 14.0, 17.0, 21.0, 26.0, and 29.0 using polynomials ($n=9$). These graphs clearly explain the modification of convergent states through changes in the shape of potential functions. It should be noted that these analyses were based on an approximation, and *PC1* extracted through PCA does not explain most of the properties of gait patterns.

The results indicate the following points:

- At $\omega$ 6.5 and 7.0, the potential functions have one stable solution around $PC1 = \pi$. The steep gradients of these potential functions suggest rapid convergence to a steady state.
- At $\omega$ 12.0 and 14.0, the potential functions are bimodal. We found another solution ($PC1=0$) and an unstable solution for each. For $\omega$ 12.0, the

convergence pattern is determined by the initial conditions; in contrast, for $\omega$ 14.0, the gait patterns nearly converged to another gait pattern (*PC1=0*) according to the shape of the potential functions. These facts are consistent with the results shown in Fig. S18. Furthermore, the bimodal potential structure clearly explains a gap in locomotion speed in the gray region in Figs. S15 (A) and (B), where another gait pattern was a low-speed gait (Movie S6).

- For $\omega$ 17.0 and 21.0, the shape of the potential functions is modified from that for $\omega$ 14.0, where we found other solutions corresponding to half-bound and canter gaits. The non-unimodal structure of these potential functions represents the weak stability of these gait patterns. This indicates “critical fluctuations” and “slow convergence” to a steady state, which are general characteristics in the transition regions in “non-equilibrium phase transition” (*31*).
- For $\omega$ 26.0 and 29.0, the shapes of the potential functions are modified from those for $\omega$ 21.0, and the solutions converged to a galloping gait. The non-unimodal structure of the potential functions for $\omega$ 29.0 represents the weak stability of the gait pattern.

Using the obtained potential functions, we visualized the phase convergence on the potential functions in Fig. S21 (Fig. 3 (B)). Here, we use the polynomials for the regression (*n=9*). The time evolution of phase convergence depending on initial conditions was in line with the shapes of the potential functions. Therefore, the potential functions can capture the dynamical structure in the gait patterns generated by our quadruped robot with the *non-wired* CPG model. In particular, the bimodal structure in the potential function at $\omega$ 12.0 and 14.0 indicates two possible steady states, depending on the initial conditions.

## 7. Reproducibility and reversibility of gait transition

To investigate the reproducibility and reversibility of gait transitions, we conducted gait transition experiments with the following eight conditions: from $\omega_1 = 7.0$ to $\omega_2 = 17.0$, from $\omega_1 = 17.0$ to $\omega_2 = 7.0$, from $\omega_1 = 7.0$ to $\omega_2 = 26.0$, from $\omega_1 = 26.0$ to $\omega_2 = 7.0$, from $\omega_1 = 9.0$ to $\omega_2 = 17.0$, from $\omega_1 = 17.0$ to $\omega_2 = 9.0$, from $\omega_1 = 9.0$ to $\omega_2 = 26.0$, and from $\omega_1 = 26.0$ to $\omega_2 = 9.0$. In these experiments, we changed the parameters as follows: $\omega=\omega_1$ during 0-10 s; $\omega$ is changed linearly from $\omega_1$ to $\omega_2$ during 10-20 s; and $\omega = \omega_2$ during 20-30 s. We conducted these experiments through more than six trials for each condition. Figures S22 (A) and (B) show the results of the above gait transition experiments.

These results indicate that gait transitions from low-speed ($\omega$= 7.0 or 9.0) to high-speed ($\omega$= 17.0 or 26.0) were successfully achieved for almost all trials. This fact strongly indicates the *reproducibility* of the gait transition experiments with our CPG model. On the other hand, we found some different features in the gait transition from high- to low-speed gait patterns. At gait transitions from $\omega = 17.0$ to 9.0, most trials showed gait transition from a half-bound to a trot, whereas at gait transitions from $\omega = 26.0$ to 9.0, some trials did not converge to a trot. These results suggest that some trials remained in another stable gait pattern in the potential structure for $\omega = 9.0$, as shown in Fig. S20. Furthermore, the timings of gait transitions were different between the increasing and decreasing of locomotion speed according to the stability difference between gaits before and after transitions, i.e. transition to a less stable gait requires a long period for convergence, e.g. $\omega = 7.0$ to 17.0. This is the reason for the nonlinear variation in $\omega$ under gait transition, as shown in Fig. 2.

Using the potential function obtained in the previous section, we visualized the dynamical modification of potential functions in the gait transition from low to high speeds and from high to low speeds of locomotion, as shown in Fig. S23. Here, we also use polynomials for regression ($n=9$). The shape modification of potential functions according to the change in $\omega$ formed *paths* for gait transitions. In the case of increased locomotion speed, unstable gait patterns were observed for a short time at $PC1_n \approx -1.0\pi$ for $\omega$=17.0. In the case of decreased speed, however, stable gait patterns were observed at $PC1_n \approx 0.0$ for $\omega$=9.0. Such differences in the gait transition path based on potential modification resulted in a "hysteresis" phenomenon.

Past biological studies have investigated the gait transitions of animals (*31, 35-37*). Gait transition in humans (*31*) and quadrupeds (*10, 38, 39*) as well as left- and right-hand coordination (*40*) and limb coordination between two people (*41*) have been analyzed from the viewpoint of "non-equilibrium phase transition" phenomena. In our model, the obtained gait patterns had a stable phase relationship and minimum energy expenditure. Moreover, gait transition occurred at the point where the system lost its stability and had to reduce its energy cost, as shown in Fig. 3 of the main text. Our study is the first experimental evidence for trot-gallop gait transition that considers the non-equilibrium phase transition of nonlinear dynamical systems, including general characteristics, e.g. "hysteresis", "critical fluctuation", and "critical slow convergence" in transition regions. This fact suggests that our model can capture the essential mechanism of leg coordination in quadruped gait transition.

## 8. Limitations of our model

Our model does not explain all observations of actual quadruped gaits because of its simplicity. Our model does not consider "intralimb coordination", i.e. coordination between joint movements in each leg, for gait generation. In particular, with changes in speed, the foot trajectory of our quadruped robot is not changed, and the stride length is changed only slightly. This is one reason why cantering and galloping in our quadruped robot did not include a sufficient suspension phase in comparison to actual quadruped animals (*61, 62*). Moreover, intralimb coordination is also essential for controlling the balance of vertical force and resulting in a sufficient Froude number. Thus, the insufficient cantering velocity (Froude number) is also a result of the lack of an intralimb coordination mechanism. In addition, our quadruped robot does not have a head component, whereas actual quadrupeds exploit head movements in gait transitions (*63, 64*).

Although our model has some limitations with respect to its ability to explain quadruped locomotion, we conclude that our model describes an essential mechanism underlying quadruped interlimb coordination based on experimental evidence. The mode reproduced the gait transition from walking to trotting to galloping without any interlimb neural connections between leg controllers.

**References**


50. Raibert, M. H., Chepponis, M., Brown, H. B. Jr. Running on four legs as though they were one. *IEEE Trans. Robot. Autom.* **2**, 70-82 (1986).

51. Herr, H. M., McMahon, T. A. A Trotting Horse Model. *IJRR* **19**, 566-581 (2000).

52. Herr, H. M., McMahon, T. A. A Galloping Horse Model. *IJRR* **20**, 26-37 (2001).

53. Weingarten, J. D., Groff, R. E., Koditschek, D. E. A framework for the coordination of legged robot gaits. *Proc IEEE RAM 2004*, Singapore (1-3 December 2004).

54. Ruina, A., Bertram, J. E. A., Srinivasan, M. J. A collisional model of the energetic cost of support work qualitatively explains leg sequencing in walking and galloping, pseudo-elastic leg behavior in running and the walk-to-run transition. *J. Theor. Biol.* **237**, 170-192 (2005).

55. Righetti, L., Ijspeert, A. J. Pattern generators with sensory feedback for the control of quadruped locomotion. *Proc. IEEE ICRA 200*8, Pasadena, CA. US. (19 to 23 May 2008).

56. Owaki, D., Morikawa, L., Ishiguro, A. Why do quadruped exhibit exclusively either trot or pace gaits? *Proc. Dynamic Walking 2013,* Pittsburgh, PA. US. (10 to 13 June 2013).

57. Spenko M. J. et al. Biologically Inspired Climiling with a Hexapodal Robot. *J. Field Robot.* **25**, 223-242 (2008).

58. Owaki, D., Morikawa, L. & Ishiguro, A. Listen to body's message: Quadruped robot that fully exploits physical interaction between legs. In *IROS2012* (2012).

59. Alexander, R. M. *Optima for Animal*, Princeton University Press (1996).

60. Zeeman, E. C. Catastrophe Theory. *Sci. Am.* **4**, 65-83 (1976).

61. Maes, L. D., Herbin, M., Hackert, R., Bels, V. L. & Abourachid, A. Steady locomotion in dogs: temporal and associated spatial coordination patterns and the effect of speed. *J Exp Biol* **211**, 138–49 (2008).

62. Biancardi, C. M. & Minetti, A. E. Biomechanical determinants of transverse and rotary gallop in cursorial mammals. *J. Exp Biol* **215**, 4144–56 (2012).

63. Minetti, A. E., Ardigo, L. P., Reinach, E. & Saibene, F. Relationship between mechanical work and energy expenditure of locomotion in horses. *J Exp Biol* **202**, 2329–2338 (1999).

64. Dunbar, D. C., Macpherson, J. M., Simmons, R. W. & Zarcades, A. Stabilization and mobility of the head, neck and trunk in horses during overground locomotion: comparisons with humans and other primates. *J Exp Biol* **211**, 3889–907 (2008).

**Table S1**. State-of-the-art of interlimb coordination in legged locomotion

| Author, year | Platform | Control model | Sensory feedback | Mechanical model | Reproduced gaits | Gait transition | Others |
|---|---|---|---|---|---|---|---|
| Raibert 1990 (*32*) | Robot | Virtual leg, forward running speed, and the pitch and roll attitude of the body (Raibert et al. 1986 (*50*)) | Angles of body, forward velocity, etc. | 3-D, whole body, rigid spine | Trot, pace, bound = 3 | Change the control algorithm (defined) | Controllers for virtual legs are decoupled, but controllers are completely coupled within virtual legs |
| Collins et al. 1994 (*6*) | Simulation | Hard-wired* CPG | None | None | Walk, trot, bound = 3 | Change more than four parameters in the oscillator model | |
| Cruse et al. 1998,2013 (*15,16*) | Simulation | WalkNet (sensory-driven chains of events) | Ground contact, posterior extreme position (PEP), leg angles, etc. | 3D stick insect | Hexapod walking, turning | Tetrapod to tripod | |
| Golubitsky et al. 1999 (*7*) | Theory | Re-wired** CPG | None | None | Walk, trot, pace, bound, pronk = 5 | Change the weights of the neural network (almost fully defined) | |
| Kimura et al. 1999 (*8*) | Robot | Re-wired CPG | Touch sensors, joint angle sensors etc. | 3-D, whole body, rigid spine | Trot, pronk, bound = 3 | None | Used two walking and running robots with different body structures |
| Herr et al. 2000 (*51*) | Simulation | Joint kinematics-based controller | Joint angle sensors | 2-D, flexible neck and bone | Trot = 1 | None | |
| Herr et al. 2001 (*52*) | Simulation | Joint kinematics-based controller | Joint angle sensors | 2-D, flexible neck and bone | Gallop = 1 | None | |
| Weingaten et al. 2004 (*53*) | Robot | Gait generator with touch-down feedback | Hip joint angle sensor (to detect touchdown) | 3-D, hexapod | Tripod (not quadruped) | None | |
| Ekeberg et al. 2005 (*13*) | Simulation | Reflex-modulated state machine | Hip angle and loading (using threshold) | 3-D, hind legs | Walk = 1 | None | |
| Ruina et al. 2005 (*54*) | Theory | Simplified collision model | None | None | Canter, pronk = 2 | None | |
| Righetti et al. 2008 (*55*) | Simulation | Re-wired CPG with sensory feedback | Touch sensors | 3-D, three quadruped robots | Walk, trot, pace, bound = 4 | Change the network topology (defined) | |
| Maufroy et al. 2010 (*33*) | Simulation | Local leg controller with sensory feedback + ascending coordination mechanism | Loading (using threshold) | 3-D, whole body, rigid spine | Walk, pace = 2 | None | |
| Aoi et al. 2013 (*10*) | Robot | Hard-wired CPG with sensory feedback | Touch sensors | 3-D, whole body, rigid spine | Walk, trot = 2 | Change the duty factor (one control parameter) | |
| Fukuoka et al. 2014 (*11*) | Simulation | Hard-wired CPG with sensory feedback and posture control | Loading and body posture | 2-D, rigid spine | L-S, D-S walk, trot, canter, gallop = 5 | Change the speed and body posture (two control parameters) | |
| Our model 2012, 2013, 2015 (*20, 56, 58*) | Robot | Non-wired*** CPG with local sensory feedback | *Continuous* loading | 3-D, whole body, flexible spine | L-S and D-S walk (*15*), trot and pace (*56*), canter, gallop = 6 | Change only a parameter $\omega$ | |

* "Hard-wired" means that the coupling in CPG is not changed to change the gait patterns. ** "Re-wired" means that the coupling in CPG should be rewired to change the gait patterns. *** "Non-wired" means that our model does not consider interlimb connections between oscillators.

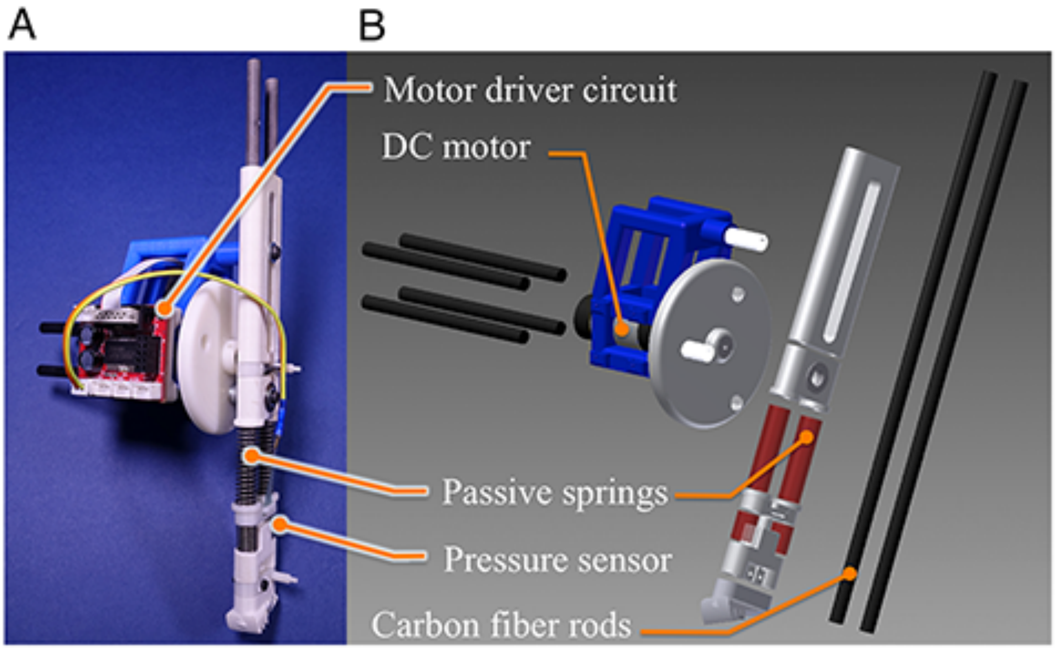


**Fig. S1 (A)** Detailed structure of the leg segment. **(B)** 3-D computer-aided design (CAD) image of leg parts. These parts consist of carbon rods and ABS resin printed on a 3-D printer.

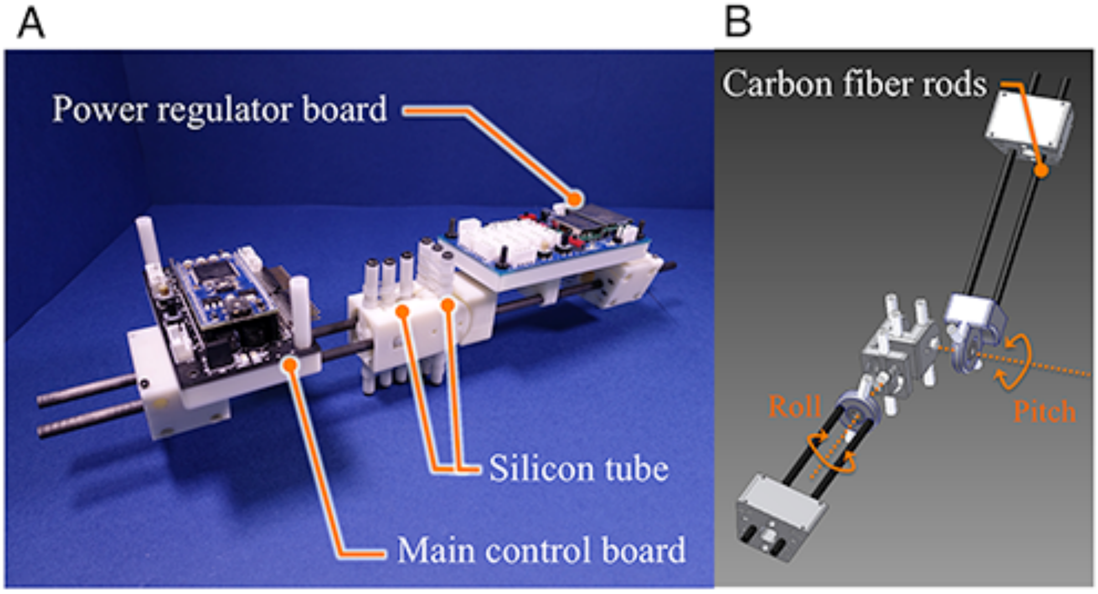


**Fig. S2 (A)** Detailed structure of backbone segment. **(B)** 3-D computer-aided design (CAD) image of backbone parts.

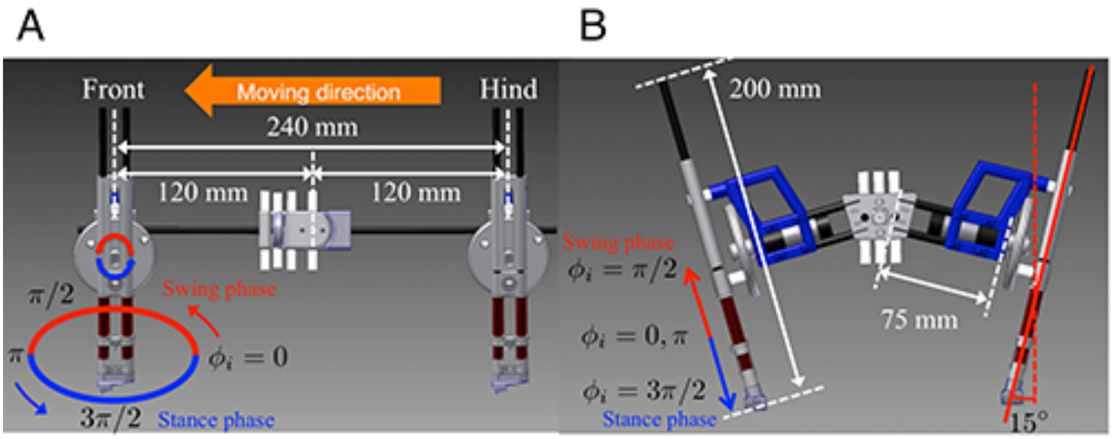


**Fig. S3** 3-D CAD image of the quadruped robot. **(A)** Side view. **(B)** Front view.

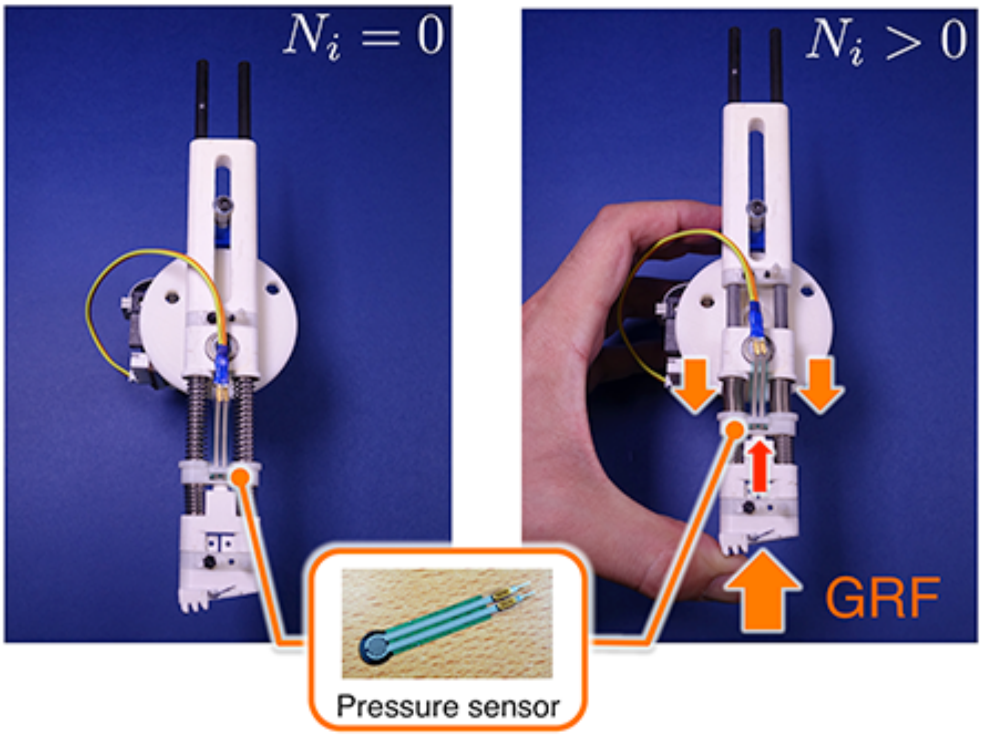


**Fig. S4** Load-sensing mechanism (Movie S8). Each foot of the robot has a pressure sensor attached to detect local ground reaction forces (GRFs).

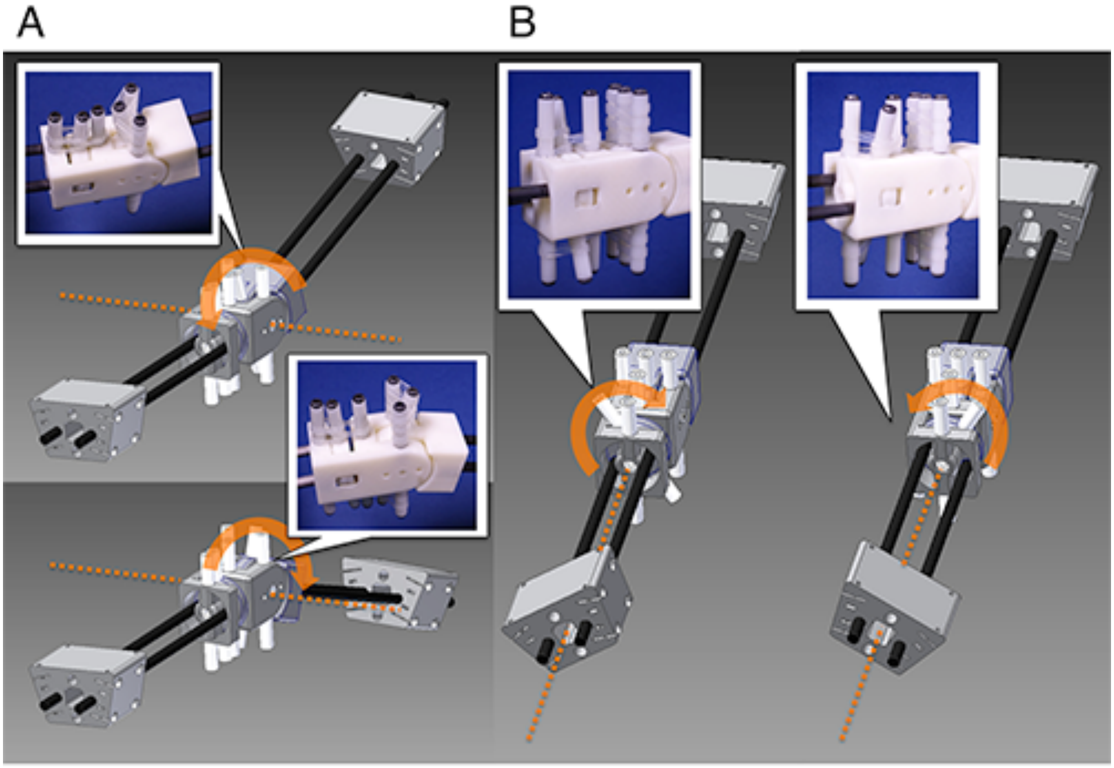


**Fig. S5** Backbone deformation mechanism using silicon tubes (Movie S9): **(A)** pitch axis and **(B)** roll axis. Profiles of the stiffness are shown in Fig. S7.

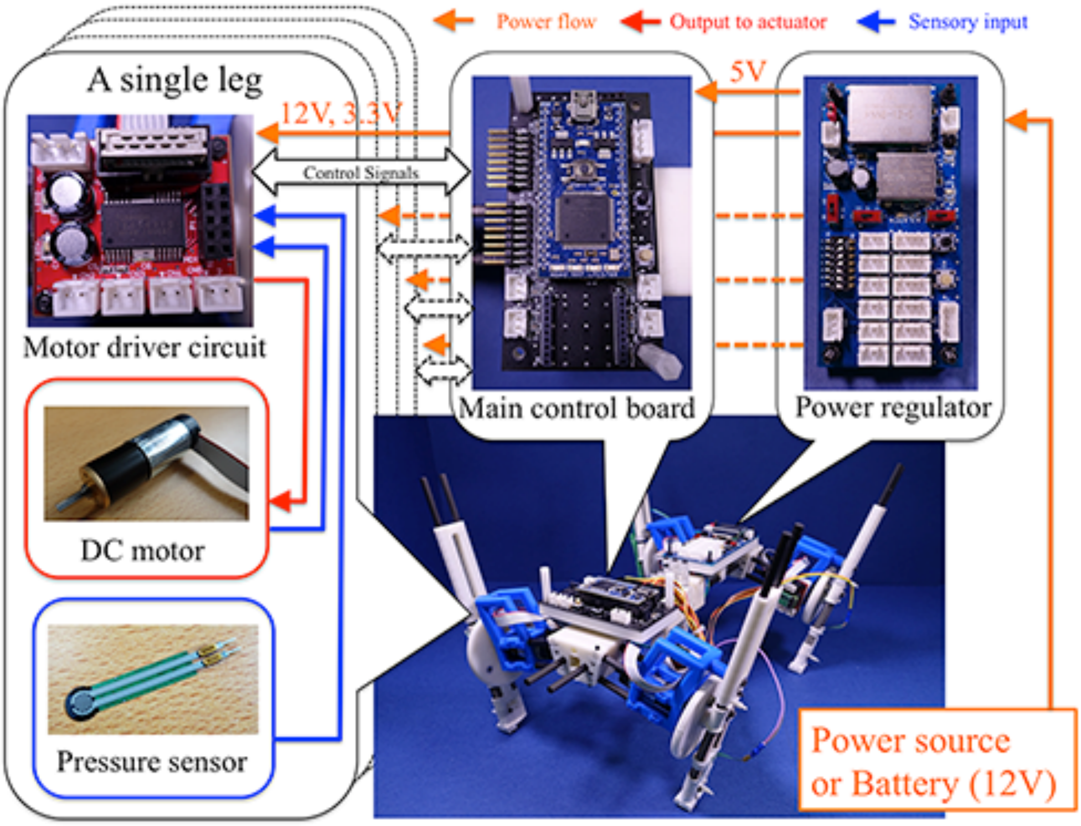


**Fig. S6** Schematic of the entire control system of our robot.

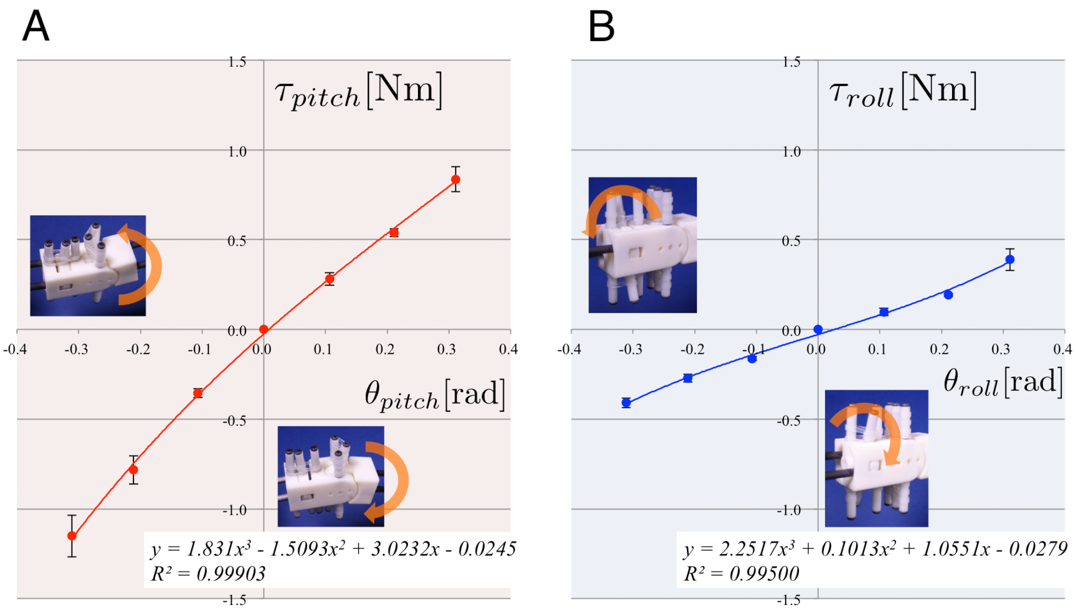


**Fig. S7** Profiles of the stiffness in the backbone joints. **(A)** Pitch and **(B)** roll axes.

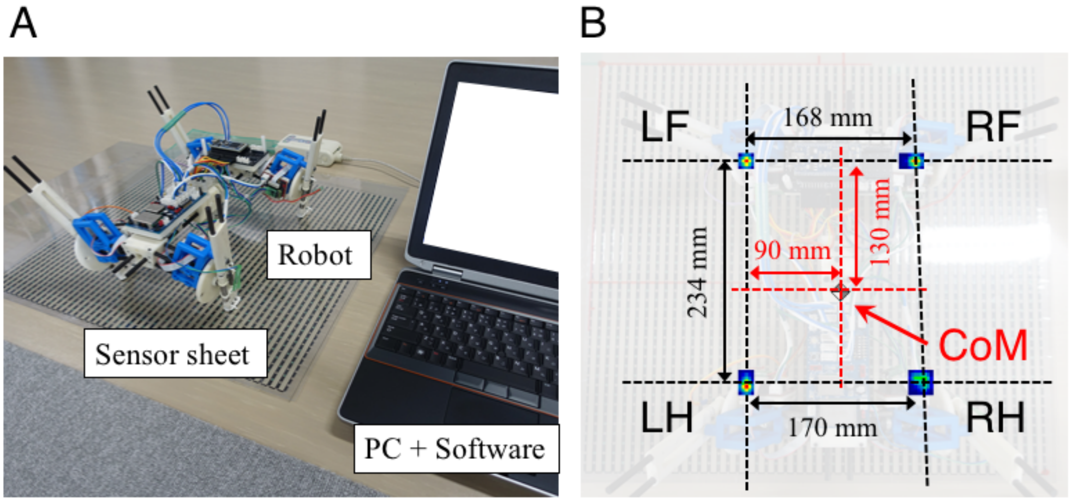


**Fig. S8 (A)** Measurement system for vertical ground forces using a pressure sensor sheet (NITTA, BIG-MAT). **(B)** Top view of our robot on the sensor sheet. The measured position of the center of mass (CoM) was close to the center of the body.

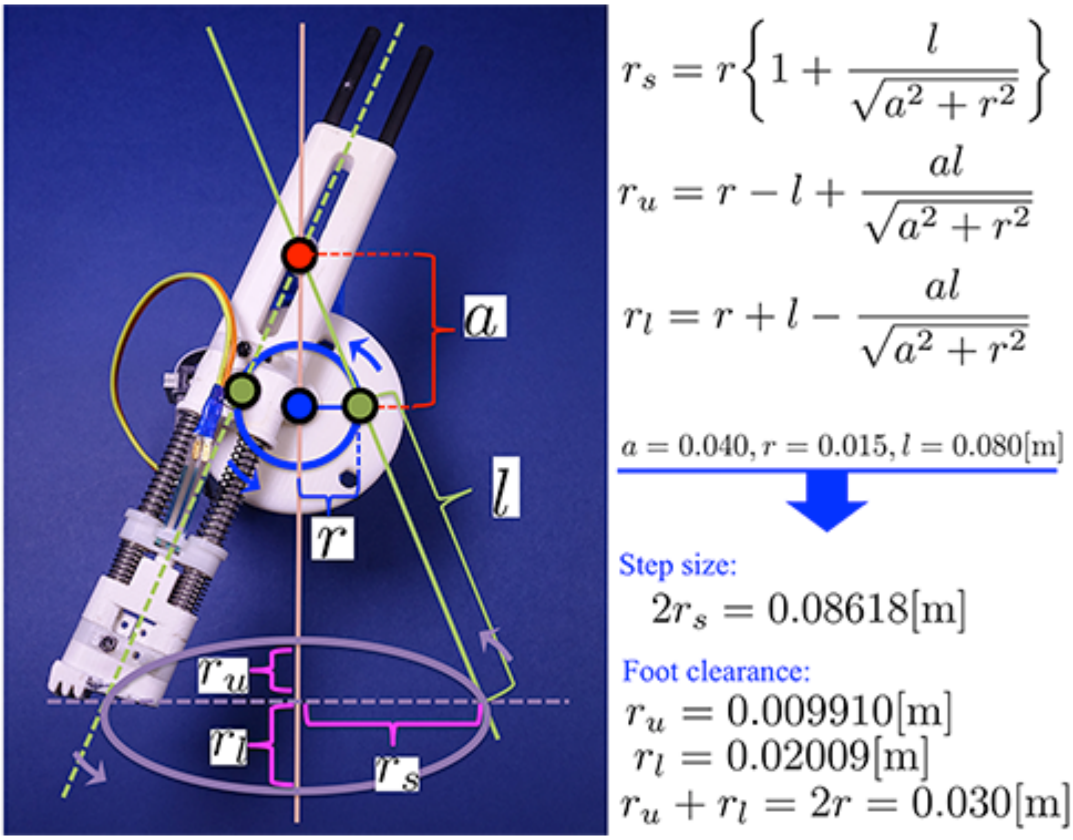


**Fig. S9** Toe trajectory of the quadruped robot. The crank mechanism converts the rotational motion of the DC motor into limb motion during the swing and stance phases. The three parameters *a, r,* and *l* determine the stride length $r_s$ and the foot clearances $r_u$ and $r_l$ of the trajectory, respectively.

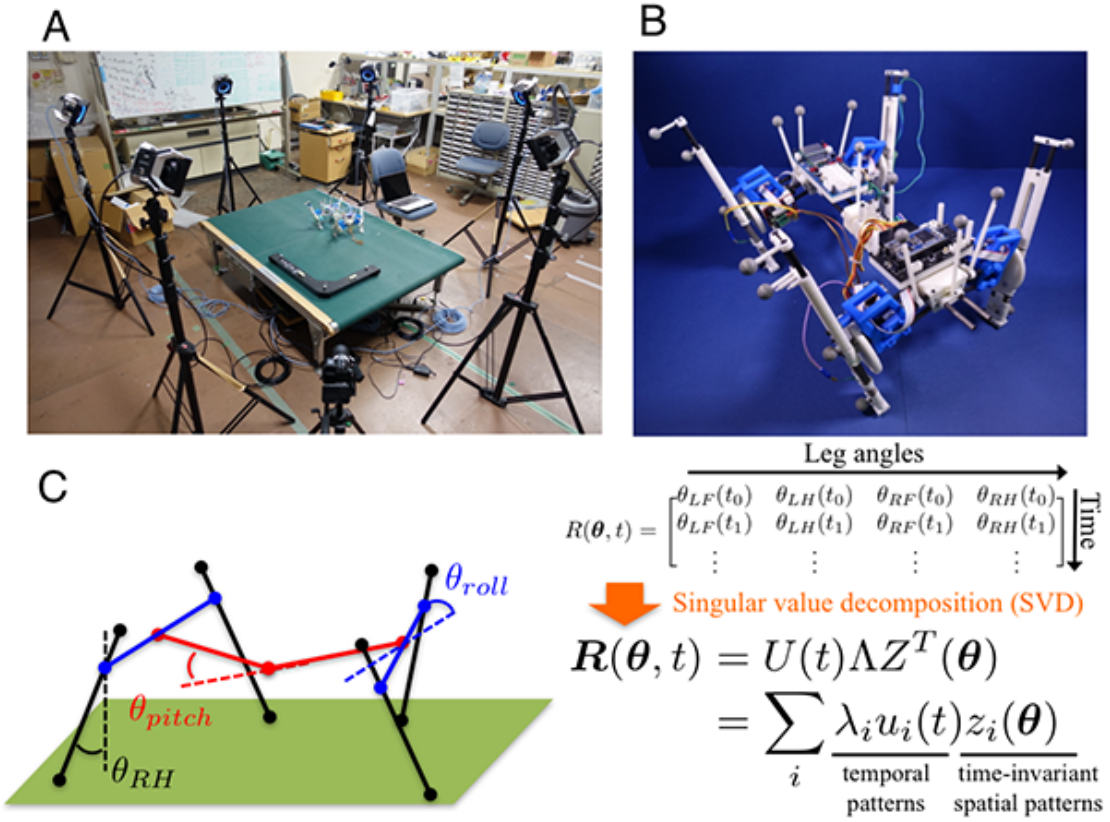


**Fig. S10 (A)** Real-time motion capture system with six high-speed cameras. **(B)** Twenty-four markers are attached to the robot (four on the fore backbone, four on the hind backbone, four on each leg) to measure real-time 3-D position data at each point. **(C)** We employed SVD to decompose time series data for each angle $R(\boldsymbol{\theta}, t)$ into spatial patterns and temporal patterns.

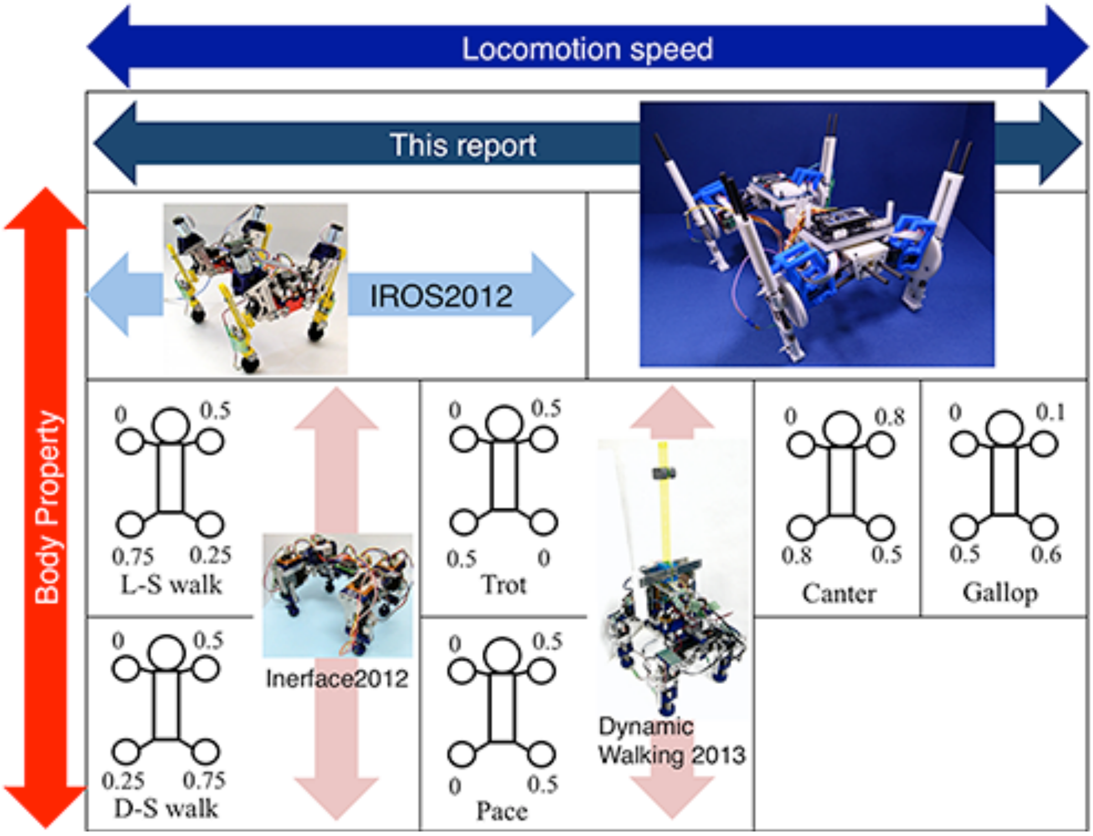


**Fig. S11** Robust gait generation demonstrated on different robotic platforms (*20, 56,* and *58*) using the same model. Regarding adaptability to changes in body properties (the vertical direction in this chart), one of our quadruped robots, *Oscillex 1,* exhibited L-S and D-S walks depending on the position of the CoM (*20*). Another of our quadruped robots, *Oscillex with a pendulum*, exhibited trot and pace gaits exclusively in cases with and without, respectively, a physical pendulum attached to its body (*56*). This study was focused on adaptability to changes in locomotion speed (the horizontal direction in this chart). The all photographs in this figure were taken by authors.

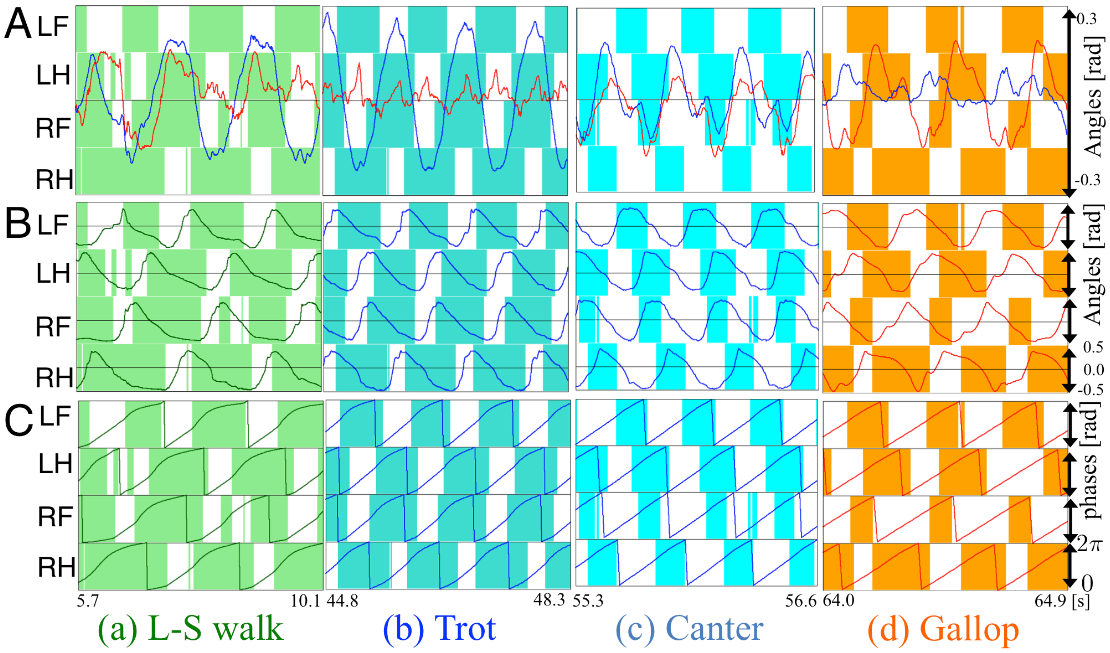


**Fig. S12** Correlation between gait diagrams, joint angles, and oscillator phases. **(A)** and **(B)** Representative kinematic data recorded by the motion capture system, shown on the gait diagrams. **(C)** Data for oscillator phases, shown on the gait diagrams. (a) L-S walk. (b) Trot with roll oscillation observed in the backbone observed. (c) Gallop with pitch oscillation observed in the backbone observed.

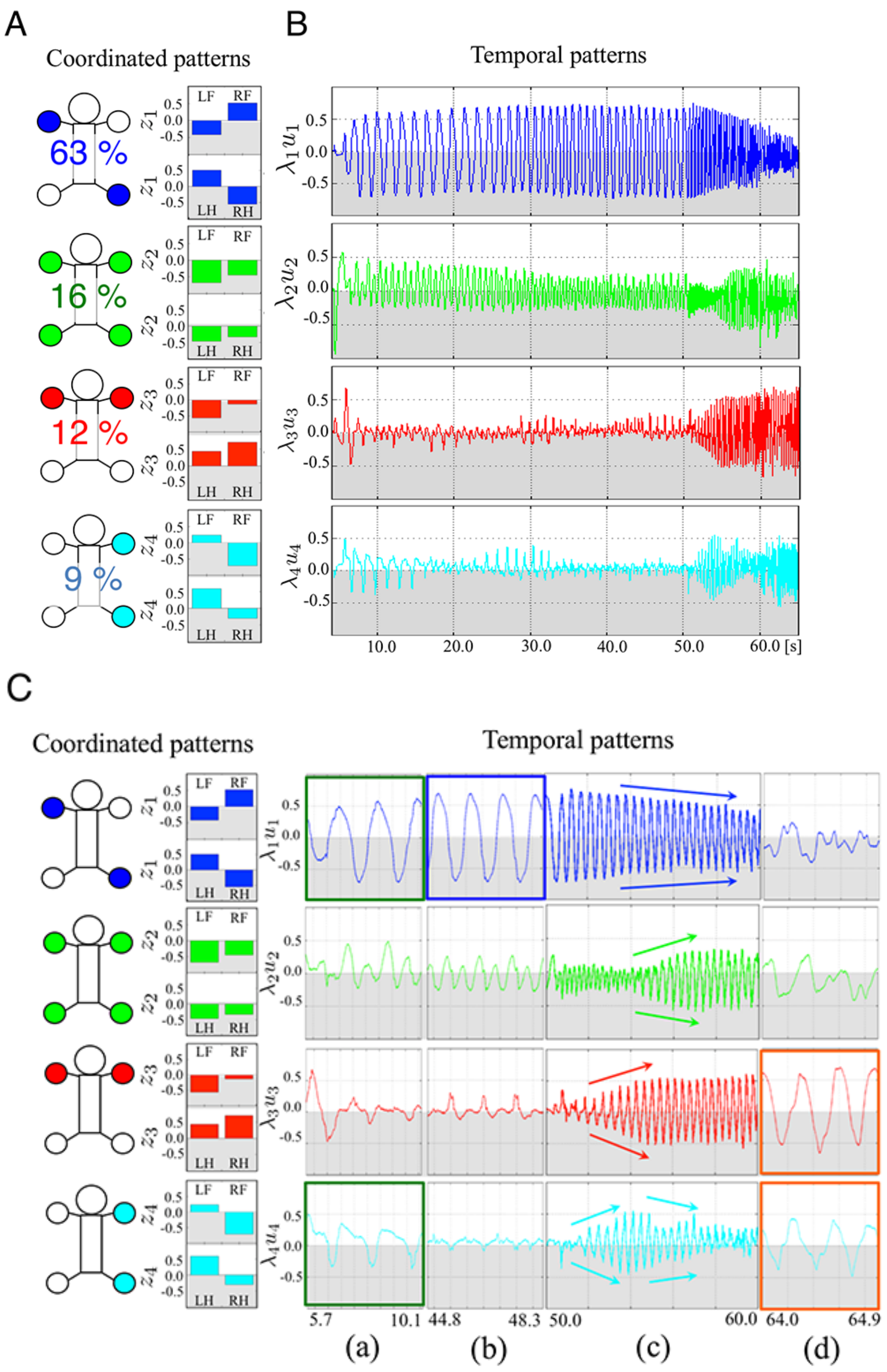


**Fig. S13** Data decomposition through SVD for four leg angles $R(\boldsymbol{\theta}, t) = [\theta_{LF}(t), \theta_{LH}(t), \theta_{RF}(t), \theta_{RH}(t)]$ converted to **(A)** spatial patterns $z_i(\boldsymbol{\theta})$ and **(B)** their temporal patterns $\lambda_i u_i(t)$ during the gait transition experiment. The data are arranged in descending order of weights of singular values $\lambda_i$, i.e. contribution ratios of patterns (shown at the extreme left) from top to bottom. (C) Highlighted data of temporal patterns for each gait (a)-(d).

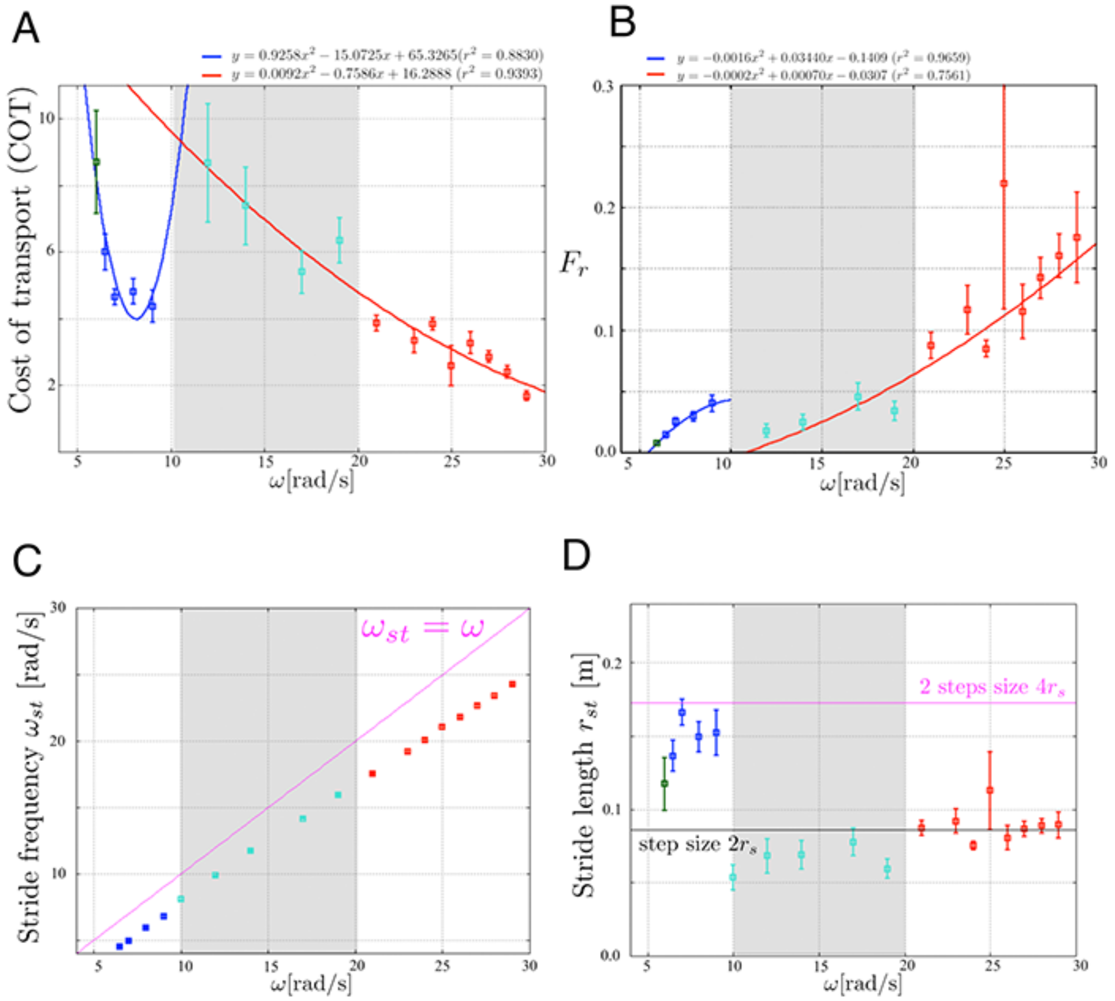


**Fig. S14 (A)** Cost of transport for each value of $\omega$ [rad/s]. The points and error bars represent the averages and standard deviations (SDs) of six trials. The colors of data points represent the range of $\omega$: dark green represents a value less than 6.5, blue represents values ranging from 7.0 to 9.0, cyan represents values between 12.0 and 19.0, and red represents values between 21.0 and 29.0.The blue and red lines represent regression curves for $\omega < 10$ and $\omega > 10$, respectively. **(B)** Froude number for each $\omega$ [rad/s]. Note that locomotion speed, i.e. the Froude number, gradually increased with $\omega$ except in the region between 10 and 12 (the gray region in B), where it decreased at one point. **(C)** Estimated stride frequency for each value of $\omega$. To calculate the stride frequency, we used the stride period $T_{st}$ [s], which is the interval between the timings when the corresponding oscillator phase is $\phi_i = \pi$ (the timing of touchdown, based on the correlation between the oscillator phases and gait diagrams in Fig. S12). $\omega_{st} = 2\pi / T_{st}$ [rad/s]. **(D)** Stride length for each value of $\omega$, estimated using $r_{st} = v \times T_{st}$.

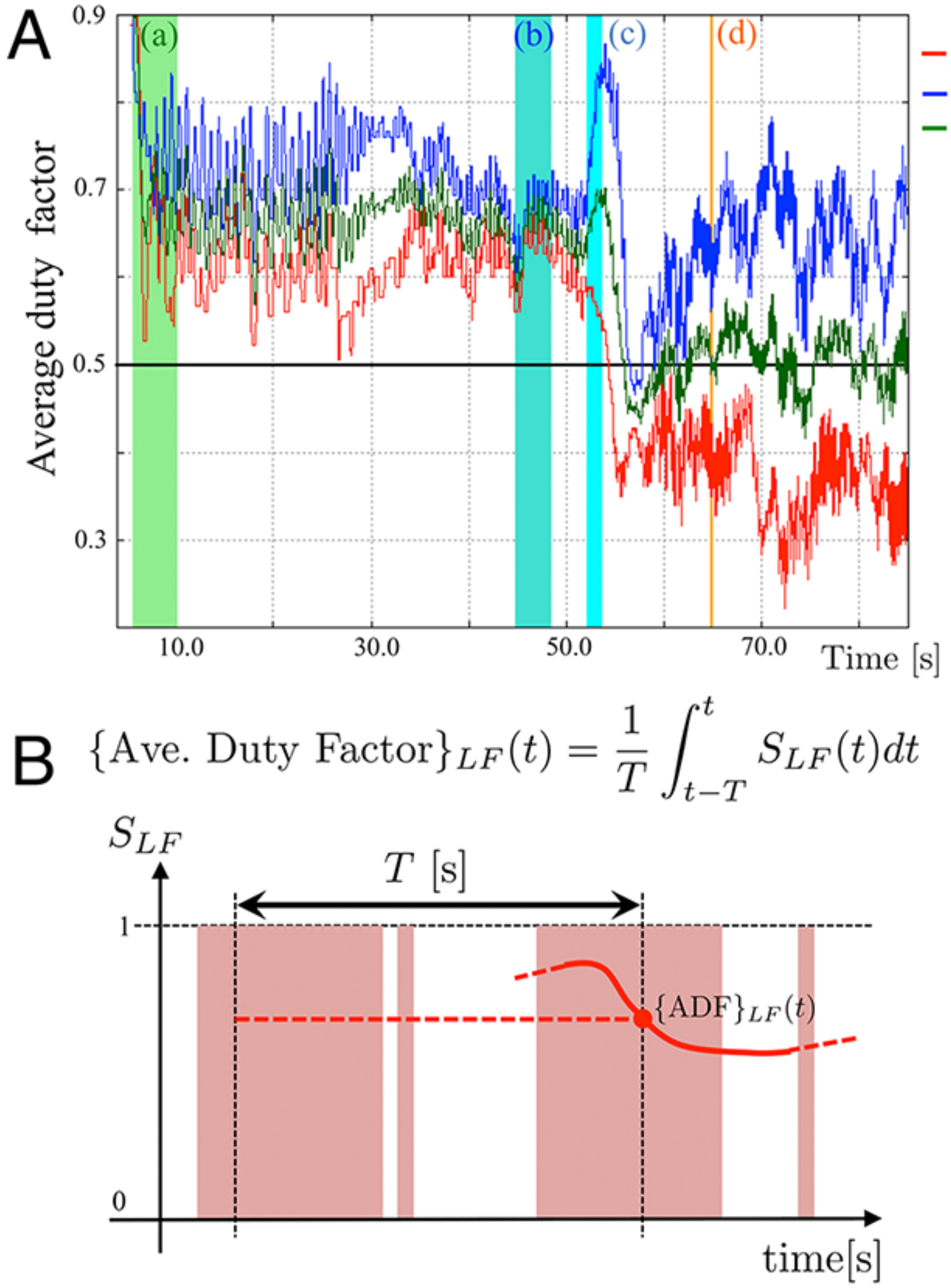


**Fig. S15 (A)** Average duty factor during the gait transition experiment. The red, blue, and green lines represent the average duty factors for the fore, hind, and all legs, respectively, over $T$ seconds, calculated using sensor value $N_i$. **(B)** Schematic procedure for the calculation of the average duty factor.

A

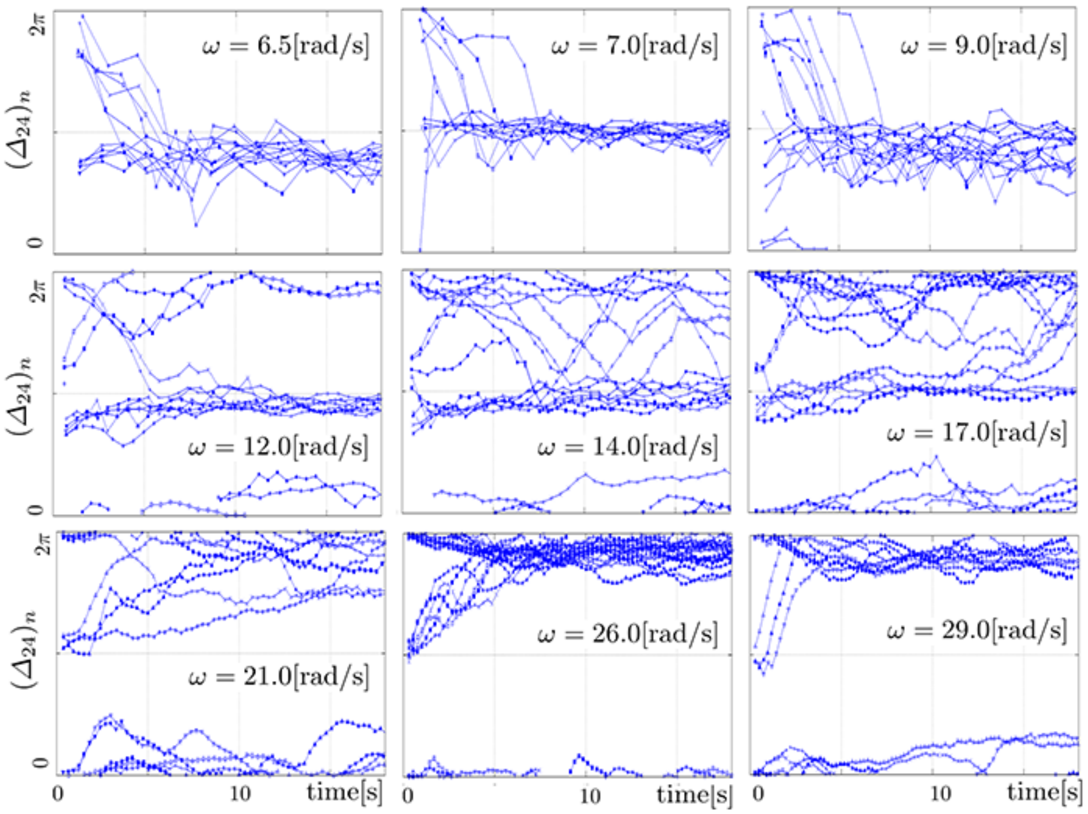


**Fig. S16 (A)** Convergence of $\Delta_{24}$, which denotes the phase difference between oscillators on the LH(2) and RH(4) legs when $\phi_1 = \pi$ for a steady state through more than 10 trials (four initial conditions) for nine patterns of $\omega$. The different markers represent the different initial conditions (trot, pace, bound, and pronk conditions).

B

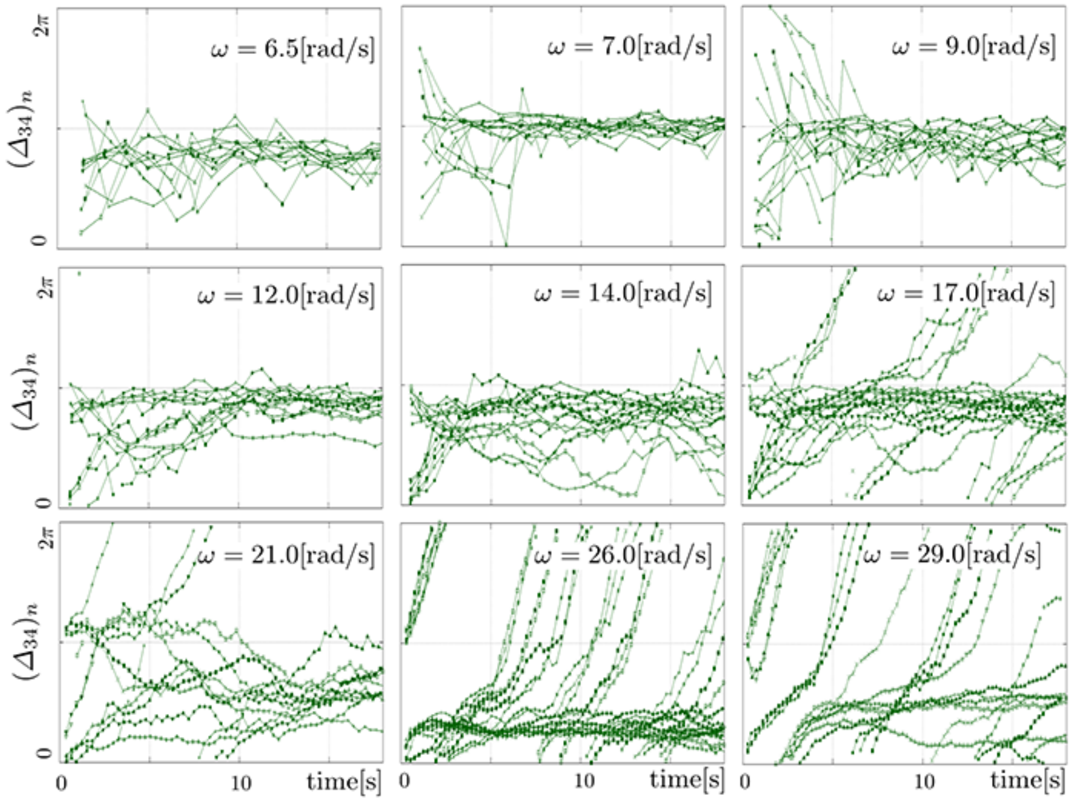


**Fig. S16 (B)** Convergence of $\Delta_{34}$, which denotes the phase difference between oscillators on the RF(3) and RH(4) legs when $\phi_1 = \pi$ for a steady state through more than 10 trials (four initial conditions) for nine patterns of $\omega$. The different markers represent the different initial conditions (trot, pace, bound, and pronk conditions).

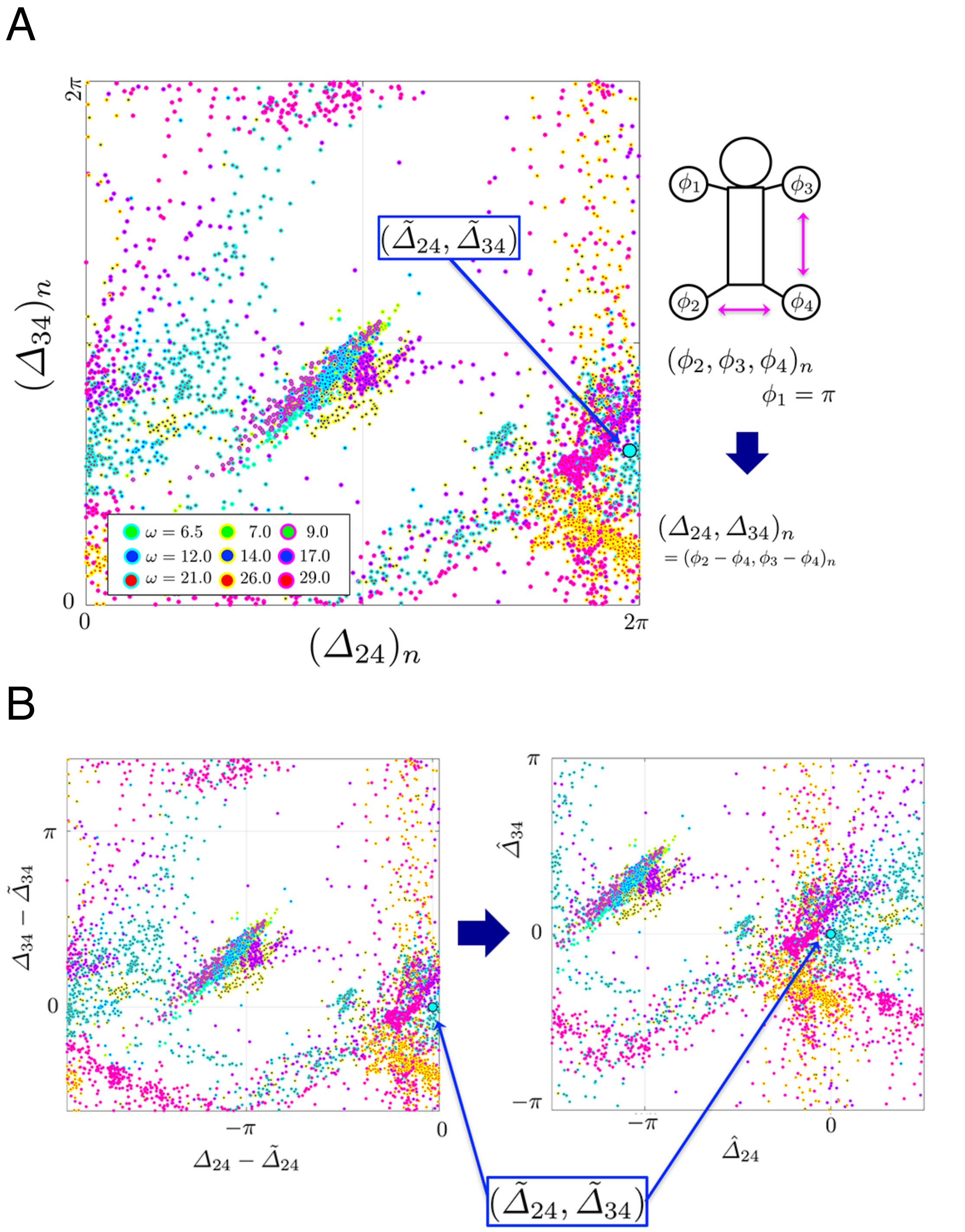


**Fig. S17** **(A)** Phase plot on the two-dimensional plane $\Delta_{24}$- $\Delta_{34}$ for various values of $\omega$. **(B)** Re-plot on the two-dimensional plane $\hat{\Delta}_{24} - \hat{\Delta}_{34}$ using average angles $(\tilde{\Delta}_{24}, \tilde{\Delta}_{34})$.

C

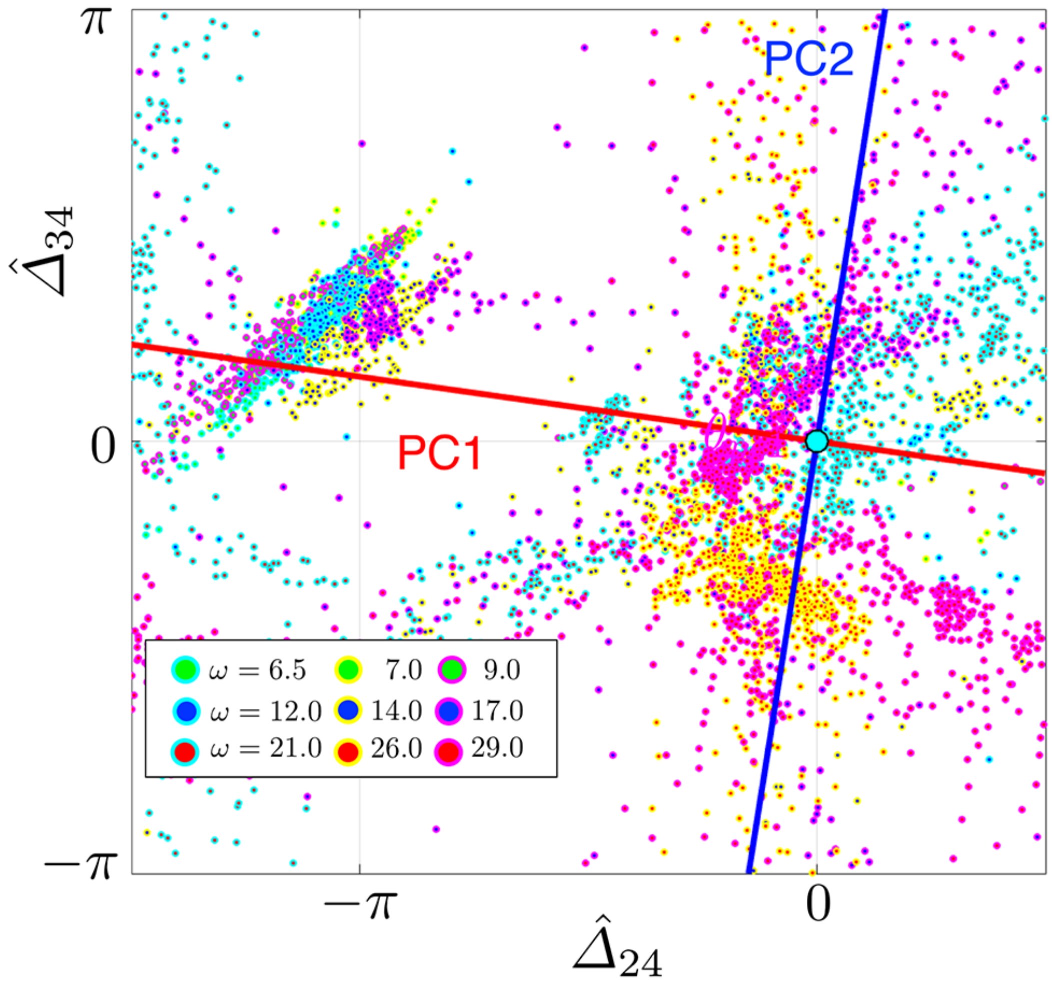


**Fig. S17 (C)** Extracting principle components for gait patterns using PCA.

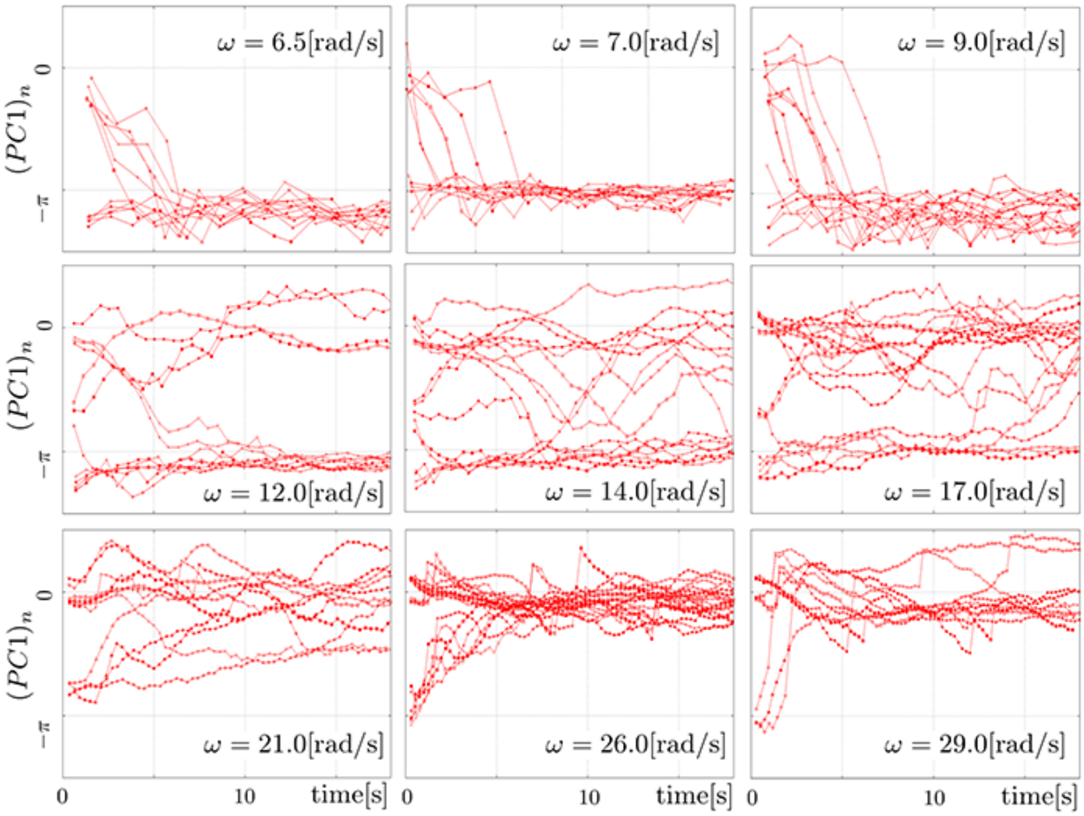


**Fig. S18** Convergence of *PC1* to a steady state for more than 10 trials for nine selected patterns of $\omega$. The different markers represent the different initial conditions (trot, pace, bound, and pronk initial conditions, respectively). These results indicate that most patterns clearly converge to one or two steady states.

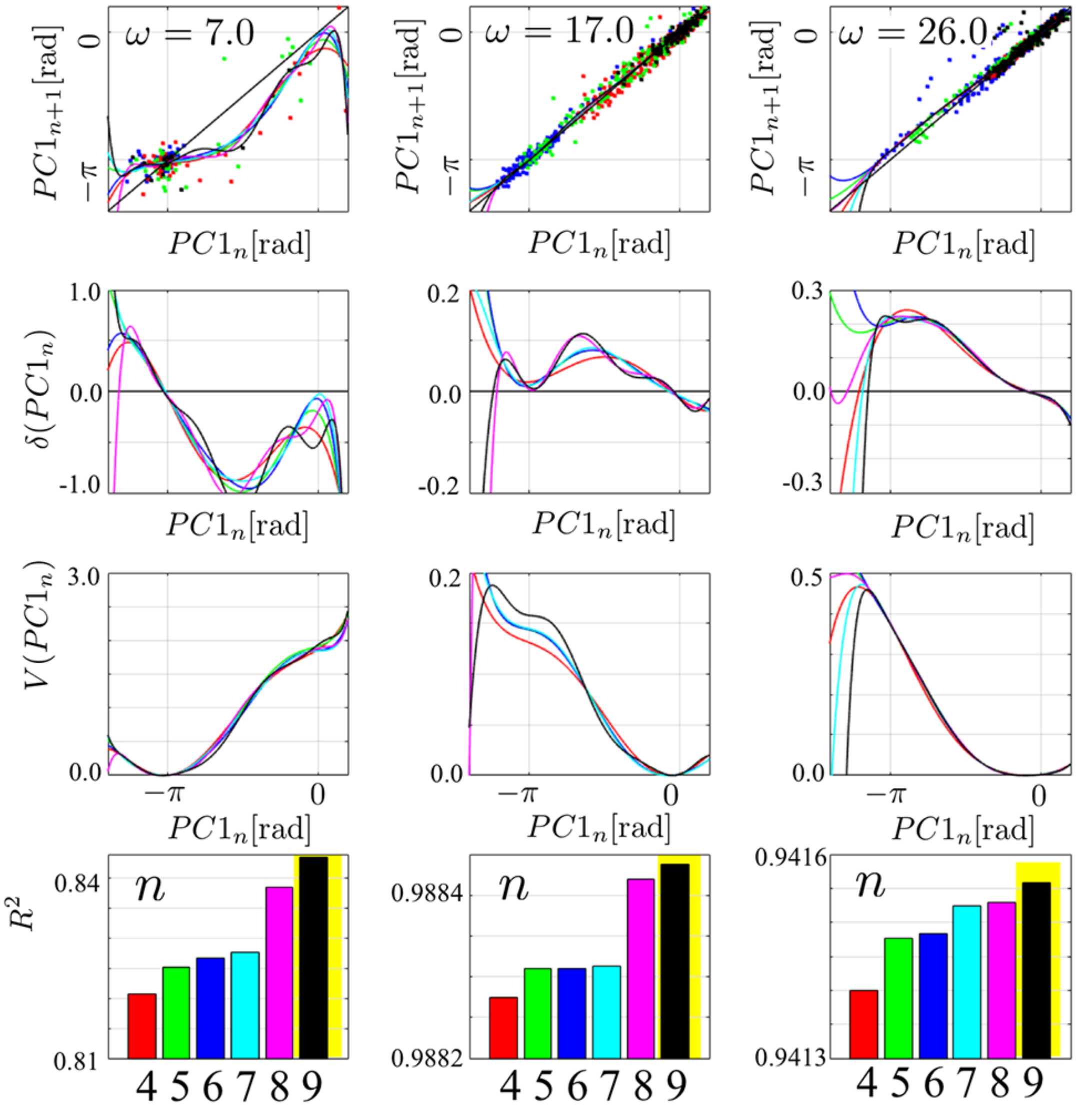


**Fig. S19** Stability analysis using return maps for $\omega$ = 7.0, 17.0, and 26.0. The graphs from the top represent return maps, $\delta_{PC1}(PC1_n)$, potential functions $V_{PC1}(PC1_n)$, and $R^2$ obtained using polynomial models for approximation.

$$PC1_{n+1} = \sum_{i=1}^{k+1} a_i (PC1_n)^{k+1-i}, \ k = 9$$

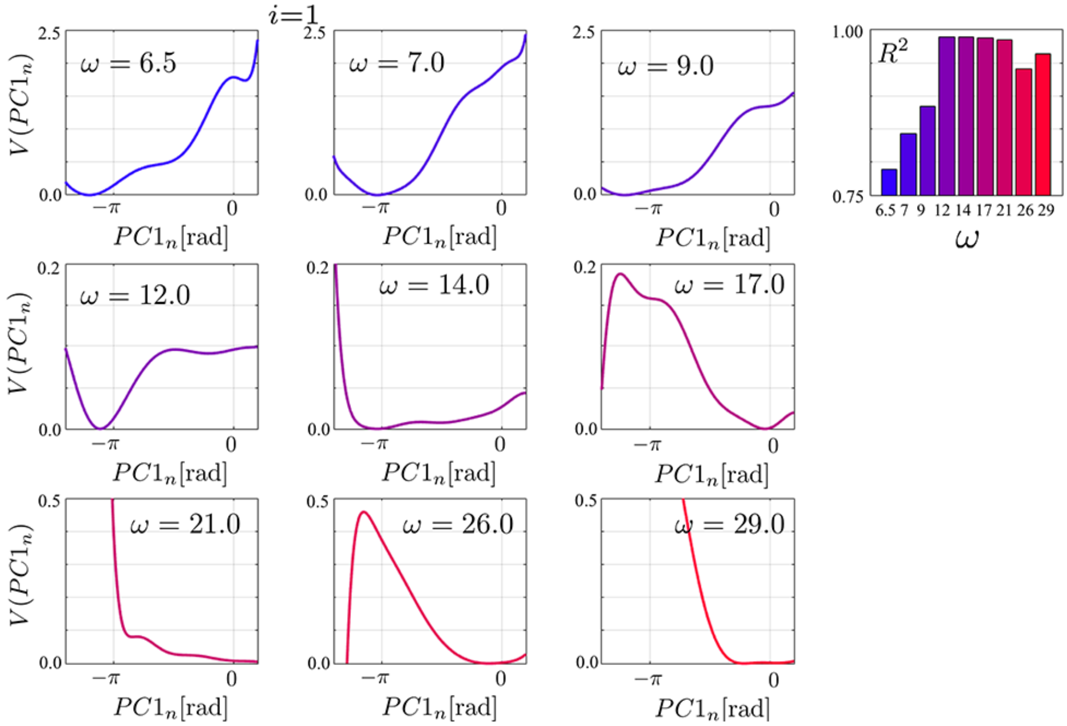


**Fig. S20** Approximate potential functions for all $\omega$ obtained using polynomials ($n$=9).

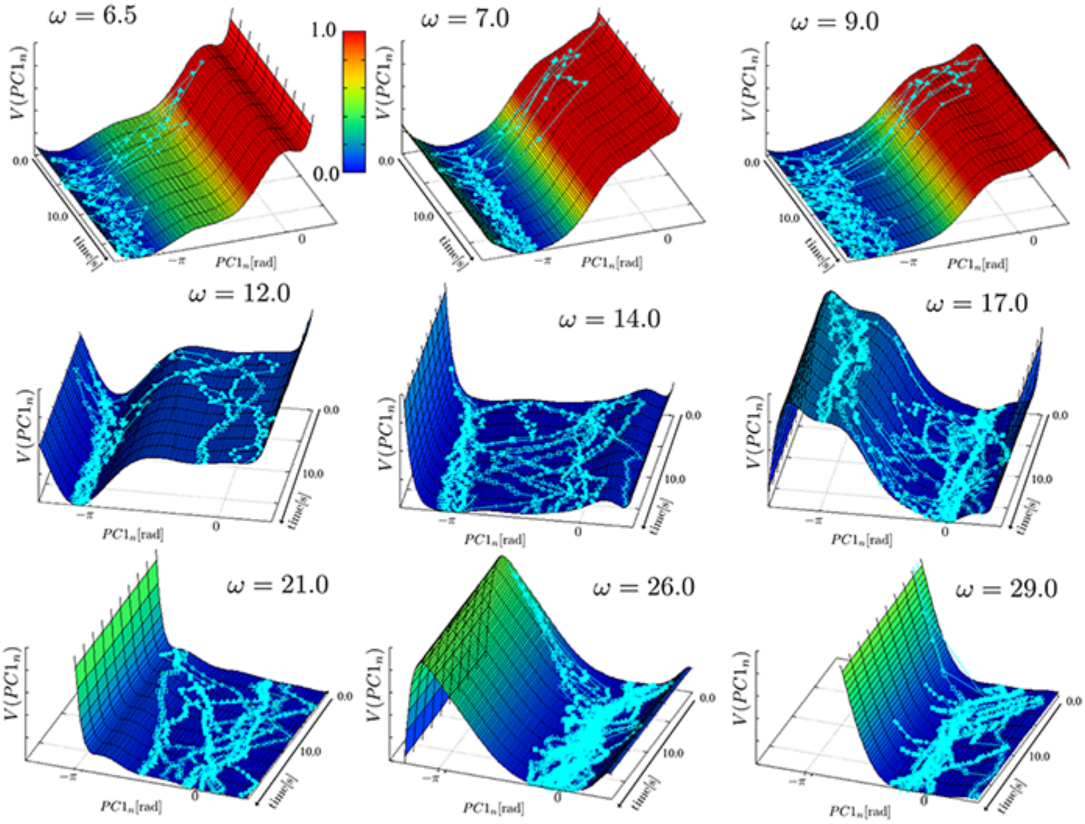


**Fig. S21** Phase convergence of $PC1_n$ on potential functions. Here, we use the polynomials ($n$=9). The time courses of phase convergence depending on initial conditions were in line with the shapes of the potential functions. In particular, bimodal structures of potential functions at $\omega$ = 12.0, 14.0, and 17.0 indicate two possible steady states depending on initial conditions.

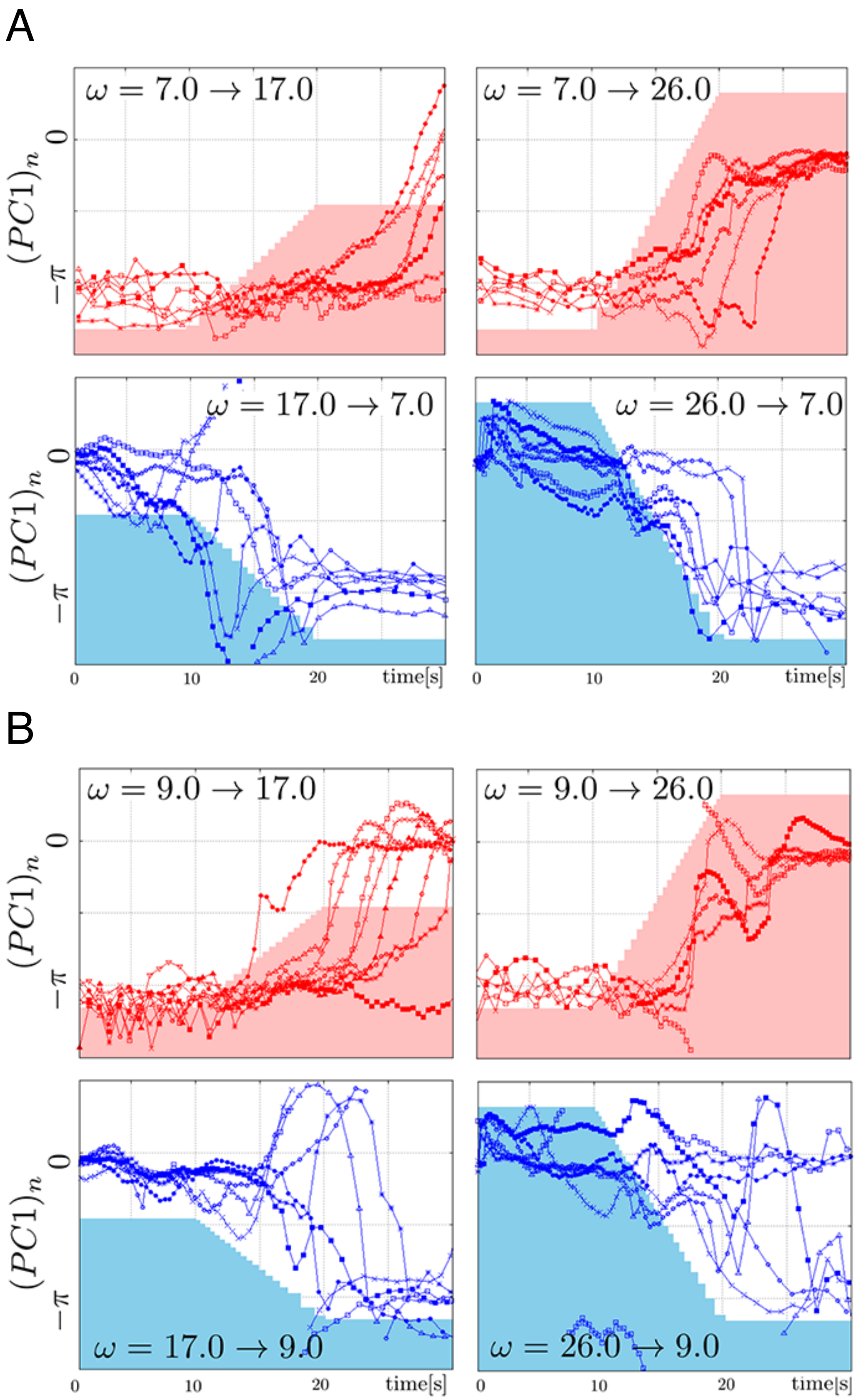


**Fig. S22** Gait transition experiments. The parameter $\omega$ is linearly changed from $\omega_1$ to $\omega_2$ during 10-20 s.

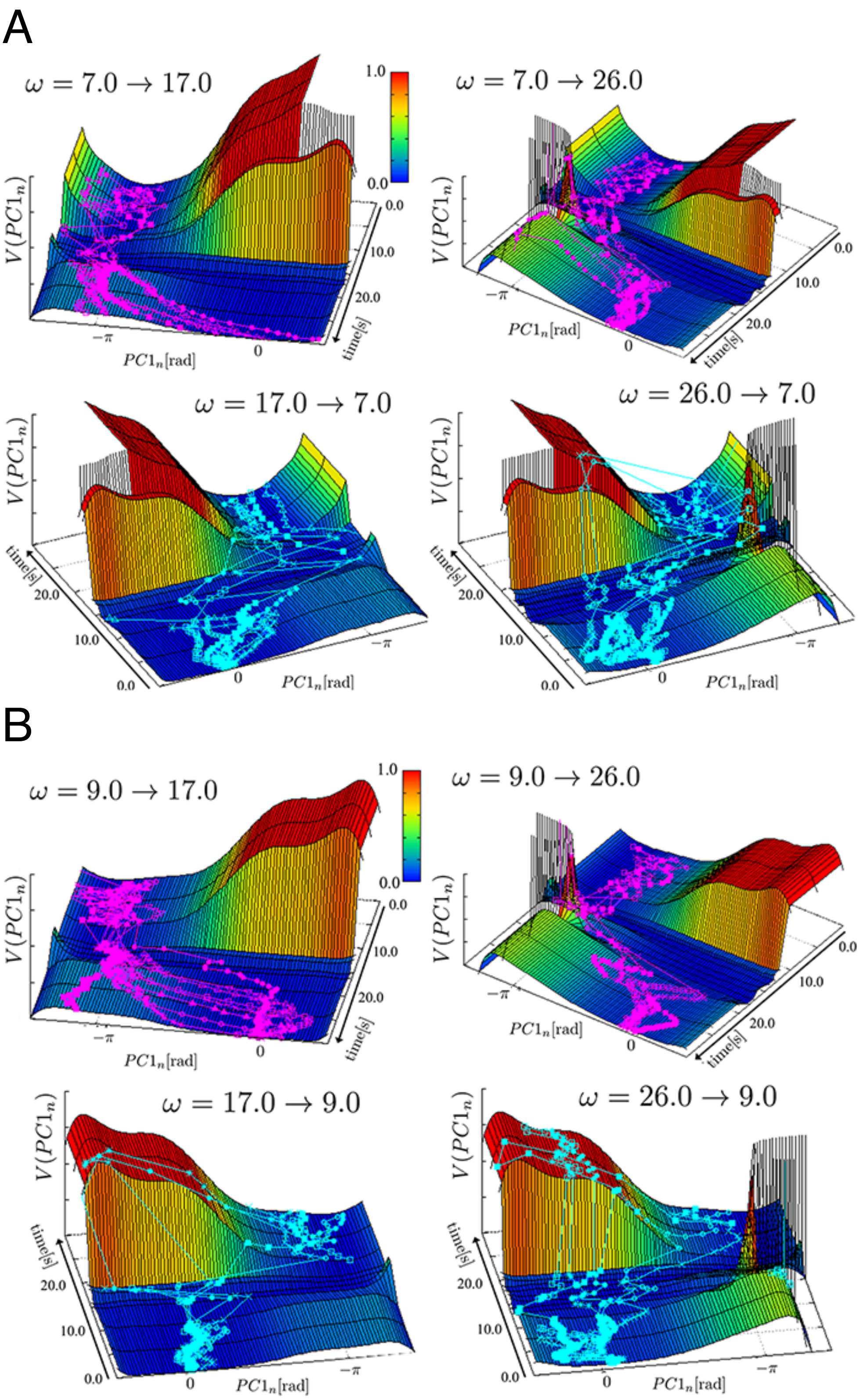


**Fig. S23** Dynamical modification of potential functions in gait transitions. Here, we use the polynomials (*n=9*). The shape modification of potential functions according to the change in $\omega$ formed *paths* for gait transitions. In particular, in the case of decreased locomotion speed (bottom figures in (B)), other stable gait patterns were observed at $PC1_n \approx 0.0$ for $\omega$ = 9.0, resulting in a "hysteresis" phenomenon.

Movie S1. Gait transition experiments with changing value of parameter $\omega$. Left: Spontaneous gait transition from L-S walk, to trot, to canter, and then to gallop was reproduced by using our CPG model. Right: Reproduced 3D motion capture data for the experiment shown in Fig. 2.

Movie S2. A gait like L-S walk for $\omega$ = 6.5 [rad/s].

Movie S3. Trot for $\omega$ = 9.0 [rad/s].

Movie S4. Gallop for $\omega$ = 26.0 [rad/s].

Movie S5. Half-bound for $\omega$ = 17.0 [rad/s].

Movie S6. Canter for $\omega$ = 21.0 [rad/s].

Movie S7. Non-convergent gait for $\omega$ = 12.0 [rad/s].

Movie S8. Leg motion in swing and stance phases, with one DC motor driving each leg according to the corresponding oscillator phases.

Movie S9. Load-sensing mechanism using passive springs and pressure sensor.

Movie S10. Passive deformation mechanism via silicon tubes along the pitch and roll axes in the backbone segment.
